# Source-resolved electroencephalogram microstates reveal opposing prefrontal-posterior dynamics and a candidate temporal-to-precuneus pathway in disorders of consciousness

**DOI:** 10.3389/fnins.2026.1995232

**Published:** 2026-10-28

**Authors:** Kun Li, Shiyu Zhang, Xu Hu, Zhigang Yan, Jijun Wang, Yongli Jiang, Feng Feng, Kexiang Gao, Xiaolong Sun, Hua Yuan

**Affiliations:** 1 Department of Rehabilitation Medicine, XiJing Hospital, Air Force Medical University (The Fourth Military Medical University), Xi’an, China; 2 School of Mechanical Engineering, Xi’an Jiaotong University, Xi’an, China; 3 Department of Rehabilitation Medicine, Shaanxi Provincial People’s Hospital, Xi’an, China; 4 Shanghai Key Laboratory of Brain-Computer Collaborative Information and Behavior, MRI Research Center, Shanghai International Studies University, Shanghai, China

**Keywords:** consciousness biomarker, disorders of consciousness, EEG microstate, Granger causality, middle temporal gyrus, precuneus, source localization

## Abstract

**Introduction:**

Objective and circuit-level biomarkers of consciousness are urgently needed for disorders of consciousness.

**Methods:**

We recorded high-density electroencephalography from 33 patients with disorders of consciousness (17 vegetative state/unresponsive wakefulness syndrome, 16 minimally conscious state) and 19 healthy controls during rest and auditory stimulation, and combined source-localized microstate analysis with hypothesis-generating Granger causality analysis.

**Results:**

Source microstate 3 (right prefrontal) and microstate 4 (parieto-occipital-temporal) showed diametrically opposite dynamics: microstate 3 was enhanced in the vegetative state whereas microstate 4 coverage and occurrence were higher in the minimally conscious state, revealing a fronto-posterior opponent imbalance. In repeated internal cross-validation within this cohort, microstate 4 occurrence discriminated the two patient groups (area under the curve = 0.812, 95% confidence interval 0.647–0.945). Hypothesis-generating connectivity analysis nominally implicated stronger right middle temporal-to-precuneus information flow in the minimally conscious state, a signal that did not survive network-level multiplicity correction.

**Discussion:**

Source-resolved microstates thus reveal opposing cortical dynamics associated with consciousness level.

## 1 Introduction

Accurate assessment of consciousness levels in patients with disorders of consciousness (DoC) remains a critical unmet need in neurocritical care and rehabilitation medicine. The Coma Recovery Scale-Revised (CRS-R) is the most widely used and validated behavioral assessment tool for DoC diagnosis, and it has substantially improved standardization and inter-rater reliability compared to earlier scales (Polyakov et al., 2025; Bodien et al., 2026). However, CRS-R remains fundamentally dependent on overt behavioral responses and is susceptible to confounding factors such as sensory impairment, motor disability, aphasia, and fluctuating arousal, resulting in misdiagnosis rates approaching 40% in clinical practice and the frequent failure to detect covert consciousness (Schnakers et al., 2009). The European Academy of Neurology has recently emphasized the urgent need for objective, complementary biomarkers to improve diagnostic accuracy and prognostic stratification in DoC (Kondziella et al., 2020). Electroencephalography (EEG) has emerged as a promising complementary objective tool owing to its high temporal resolution, portability, and suitability for bedside monitoring (Edlow et al., 2021; Zhang et al., 2025). Indeed, a recent multicenter study of 583 patients with DoC successfully translated EEG-derived features and routine clinical indicators into an explainable, externally validated machine-learning model for individualized consciousness-level diagnosis, which has been deployed as a web-based tool to support clinical decision-making (Mou et al., 2026). Nevertheless, the EEG features employed by such models—much like conventional indices such as spectral power density, entropy, or complexity measures—remain scalp-level, single-dimensional characterizations. They describe whether brain function is abnormal, but not which neural circuits are compromised, and therefore offer limited guidance for circuit-targeted therapeutic intervention.

EEG microstate analysis has advanced beyond single-dimensional metrics by capturing transient, whole-brain topographical patterns that index large-scale network dynamics (Khanna et al., 2015; Manasova et al., 2025). Compared to conventional spectral power density or linear complexity measures, microstate parameters offer greater specificity for differentiating consciousness states in DoC, as they reflect the spatial configuration and temporal evolution of distributed neural networks rather than isolated frequency components (Tarailis et al., 2024; Xu et al., 2025; Li et al., 2024; Toplutaş et al., 2024; Chen et al., 2022). However, the vast majority of microstate studies in DoC have operated at the sensor level, describing scalp potential topographies without identifying their cortical generators. Consequently, these approaches reveal that neural network dynamics differ across consciousness levels, but cannot specify where the critical dysfunctions originate or which pathways are disrupted—information essential for both mechanistic understanding and the design of targeted neuromodulation strategies.

Source localization techniques, particularly exact low-resolution electromagnetic tomography (eLORETA), have extended microstate analysis from the sensor surface to the cortical source space, enabling identification of the neuroanatomical origins of each microstate (Zhang et al., 2023; Pascual-Marqui et al., 2011; Jatoi et al., 2014; Zhang et al., 2025). Recent studies have also demonstrated that auditory paradigms can probe language processing depth in DoC patients (Gui et al., 2020), and that auditory tasks modulate EEG microstate temporal dynamics (D’Croz-Baron et al., 2021). However, these lines of investigation have proceeded largely independently. Source-level microstate studies have predominantly employed resting-state paradigms (Han et al., 2025), which capture task-negative endogenous neural activity without actively engaging the sensory processing and cognitive integration mechanisms thought to underpin conscious experience (Northoff et al., 2023). Auditory paradigm studies, in turn, have not integrated source-level localization or directed connectivity estimation to identify the specific cortical circuits mediating consciousness-level differences. Consequently, a method that unites multi-condition auditory paradigms with source-level microstate localization and directed functional connectivity analysis remains to be established. EEG-based approaches have demonstrated particular promise for bedside implementation in neurocritical care settings (Rossi Sebastiano et al., 2021). They offer a balance between diagnostic yield and clinical feasibility that neuroimaging modalities often cannot match.

Two influential theories of consciousness—the Global Neuronal Workspace Theory (GNWT) and the Integrated Information Theory (IIT)—converge on a central principle: conscious experience depends on the capacity of brain networks to transmit and integrate information across distributed regions (Mashour et al., 2020; Baars, 2005; Tononi et al., 2016). The GNWT emphasizes prefrontal-parietal broadcasting, while the IIT emphasizes posterior cortical integration; jointly, they imply that disorders of consciousness may involve the disruption of long-range pathways connecting sensory processing regions to higher-order integration hubs. We therefore hypothesize that the functional integrity of directed information transmission from auditory-related temporal cortices to posterior cortical integration hubs constitutes a critical circuit-level biomarker distinguishing distinct consciousness levels.

Based on this integrative framework, we propose two *a priori* hypotheses. First, source-level microstate analysis will reveal dissociable spatiotemporal signatures in the prefrontal cortex and posterior parieto-occipito-temporal regions that distinguish VS/UWS from MCS patients. Second, under multi-condition auditory stimulation, the directed functional connectivity from auditory-related temporal cortices to posterior cortical integration hubs will be significantly stronger in MCS than in VS/UWS, reflecting preserved bottom-up sensory integration as a neural substrate of residual consciousness. To guard against optimistic performance estimates, multivariable discriminative performance of candidate microstate markers was evaluated using cross-validated models, and connectivity analyses over a prespecified set of directed connections were treated as hypothesis-generating. Finally, we prioritized parsimonious markers, explicitly defined multiplicity-correction families, and transparent reporting over model complexity, while recognizing the limitations of internal validation in a small cohort. To test these hypotheses, we enrolled 33 DoC patients (VS/UWS = 17, MCS = 16) and 19 healthy controls. High-density EEG was acquired under resting-state and three auditory conditions (native speech, non-native speech, and white noise), followed by sequential sensor-level microstate analysis, eLORETA source localization, and frequency-domain Granger causality (GC) analysis.

The two hypotheses were prespecified to be tested by two complementary, hierarchically organized analyses. Source-level microstate analysis served as the primary method and carries all diagnostic-level claims of this study: it tests whether consciousness-related whole-brain network states differ between VS/UWS and MCS (Hypothesis 1). Granger causality analysis served as a secondary, explicitly hypothesis-generating method: it probes whether directed information flow among the cortical regions implicated by the primary analysis differs between groups (Hypothesis 2), and is not intended to provide additional diagnostic value. This sequential design mirrors the logic of the hypotheses themselves—first identifying which large-scale network states are altered, then asking how information is transmitted among the regions that define those states. This study aims to advance beyond descriptive biomarkers toward a candidate circuit-level assessment framework that, pending external validation, could inform both objective diagnosis and precision neuromodulation targeting in severely brain-injured patients.

## 2 Materials and methods

### 2.1 Participants

The study protocol was approved by the Ethics Committee of Xijing Hospital (approval number: KY20252193-X-3), which served as the central ethics review body for this multi-center study, and was conducted in accordance with the Declaration of Helsinki. All healthy controls provided written informed consent, and informed consent for DoC patients was obtained from their legal guardians/close relatives. A total of 54 participants were enrolled; 52 (33 DoC + 19 HC) completed the protocol and were included in the final analysis DoC patients were classified into the VS/UWS group and the MCS group based on CRS-R assessment (Giacino et al., 2004). CRS-R was administered 5 times by same physician within 1 week of the EEG recording. For VS/UWS group, *n* = 17, 11 males, 6 females, aged 33–69 years, mean 50.6 ± 10.9 years; For MCS group, *n* = 16 (initially 18 were enrolled, but 2 were excluded for failure to complete the experimental protocol), 8 males, 8 females, aged 23–72 years, mean 54.2 ± 13.2 years. All patients were native Chinese speakers and had not received sedative medications within 24 h prior to recording. Patient etiologies included traumatic brain injury (TBI) in 16 cases (48.5%) and stroke in 17 cases (51.5%), with disease duration ranging from 1 to 12 months (mean 4.8 ± 3.5 months). The healthy control group comprised 19 individuals (12 males, 7 females), aged 21–61 years (mean 36.6 ± 13.4 years). Demographic and clinical characteristics of the three groups are summarized in Table 1.

**Table 1:** Patient demographic and clinical characteristics.

Patient	Age (years)	Sex	Etiology	Disease duration (months)	CRS-R score	Diagnosis
1	61	M	TBI	4	13(2–1–5-2-1-2)	MCS
2	62	F	TBI	1.5	18(3–4–5-2-1-3)	MCS
3	46	M	TBI	1.5	3(0–1–1-0-0-1)	VS/UWS
4	57	M	Stroke	2.5	6(1–1–2-0-0-2)	VS/UWS
5	40	F	Stroke	4.5	7(1–3–0-1-0-2)	MCS
6	49	F	TBI	6.5	13(2–2–4-1-1-3)	MCS
7	72	M	Stroke	1.5	8(1–3–1-1-0-2)	MCS
8	68	F	Stroke	3.5	8(2–3–1-0-0-2)	MCS
9	33	F	TBI	2	6(1–1–2-0-0-2)	VS/UWS
10	45	M	Stroke	2	5(1–1–1-0-0-2)	VS/UWS
11	68	F	TBI	3	4(1–0–1-1-0-1)	VS/UWS
12	35	M	TBI	10	8(1–1–3-1-0-2)	MCS
13	64	M	TBI	1.5	12(2–1–5-2-0-2)	MCS
14	57	F	Stroke	2.5	11(1–2–5-1-0-2)	MCS
15	23	F	TBI	6	13(2–3–5-1-0-2)	MCS
16	40	F	TBI	7	7(2–1–1-1-0-2)	VS/UWS
17	69	F	Stroke	2	4(1–0–1-1-0-1)	VS/UWS
18	54	M	TBI	6	7(2–1–2-1-0-1)	VS/UWS
19	64	F	Stroke	3	5(1–0–1-1-0-2)	VS/UWS
20	55	M	Stroke	12	5(1–1–1-0-0-2)	VS/UWS
21	49	M	Stroke	7	8(2–1–2-1-0-2)	VS/UWS
22	35	F	TBI	9	4(1–0–1-1-0-1)	VS/UWS
23	57	F	Stroke	4	12(1–3–5-1-0-2)	MCS
24	59	M	TBI	4	4(0–0–1-1-0-2)	VS/UWS
25	57	M	Stroke	12	10(1–1–5-1-0-2)	MCS
26	43	M	Stroke	1	13(2–3–5-1-0-2)	MCS
27	50	M	Stroke	7	6(0–1–2-1-0-2)	VS/UWS
28	49	M	Stroke	11	6(1–0–2-1-0-2)	VS/UWS
29	55	F	Stroke	12	13(1–3–5-2-0-2)	MCS
30	67	M	TBI	1.5	12(2–3–3-2-0-2)	MCS
31	51	M	Stroke	4	4(0–0–1-1-0-2)	VS/UWS
32	36	M	TBI	1	4(0–0–1-1-0-2)	VS/UWS
33	57	M	TBI	1.5	12(1–3–5-1-0-2)	MCS

M, Male; F, Female; TBI, Traumatic brain injury; CRS-R, Coma recovery scale-revised, CRS-R subscales: auditory; visual; motor; verbal; communication; arousal. Numbers in parentheses in the CRS-R column represent subscale scores in the following order: auditory, visual, motor, verbal, communication, and arousal. MCS, minimally conscious state; VS/UWS, vegetative state/unresponsive wakefulness syndrome.

Inclusion criteria for DoC patients were: consciousness impairment lasting 28 days to 1 year after brain injury, normal auditory function prior to illness, and no history of pre-existing psychiatric or neurological disorders (such as neurodegenerative diseases or malignant brain tumors). Exclusion criteria included unstable vital signs, active epilepsy, severe skull/scalp defects, and current use of sedative medications. Inclusion criteria for healthy controls were: physical health, normal hearing, and no history of psychiatric or neurological disorders. All data were de-identified prior to analysis to ensure confidentiality.

### 2.2 Experimental design

This study employed three types of auditory stimuli: Chinese (Mandarin) speech, Spanish speech, and white noise. Speech materials were derived from public-domain audiobooks, containing multiple male and female speakers to minimize the influence of individual voice characteristics or speech rate. All audio files were manually screened to remove background noise and silence at the beginning or end. Files were standardized using Adobe Audition (Adobe Systems) to a 44.1 kHz sampling rate, 16-bit depth, and dual-channel WAV format. The amplitude of all segments was normalized (root-mean-square energy or loudness normalization) to ensure consistent volume without compromising intelligibility. White noise was generated using MATLAB (MathWorks) with identical sampling parameters and duration.

The experiment employed a block design comprising three identical blocks (Figure 1). Each block contained nine audio segments (1 min each): three Chinese segments, three.

**Figure 1:**
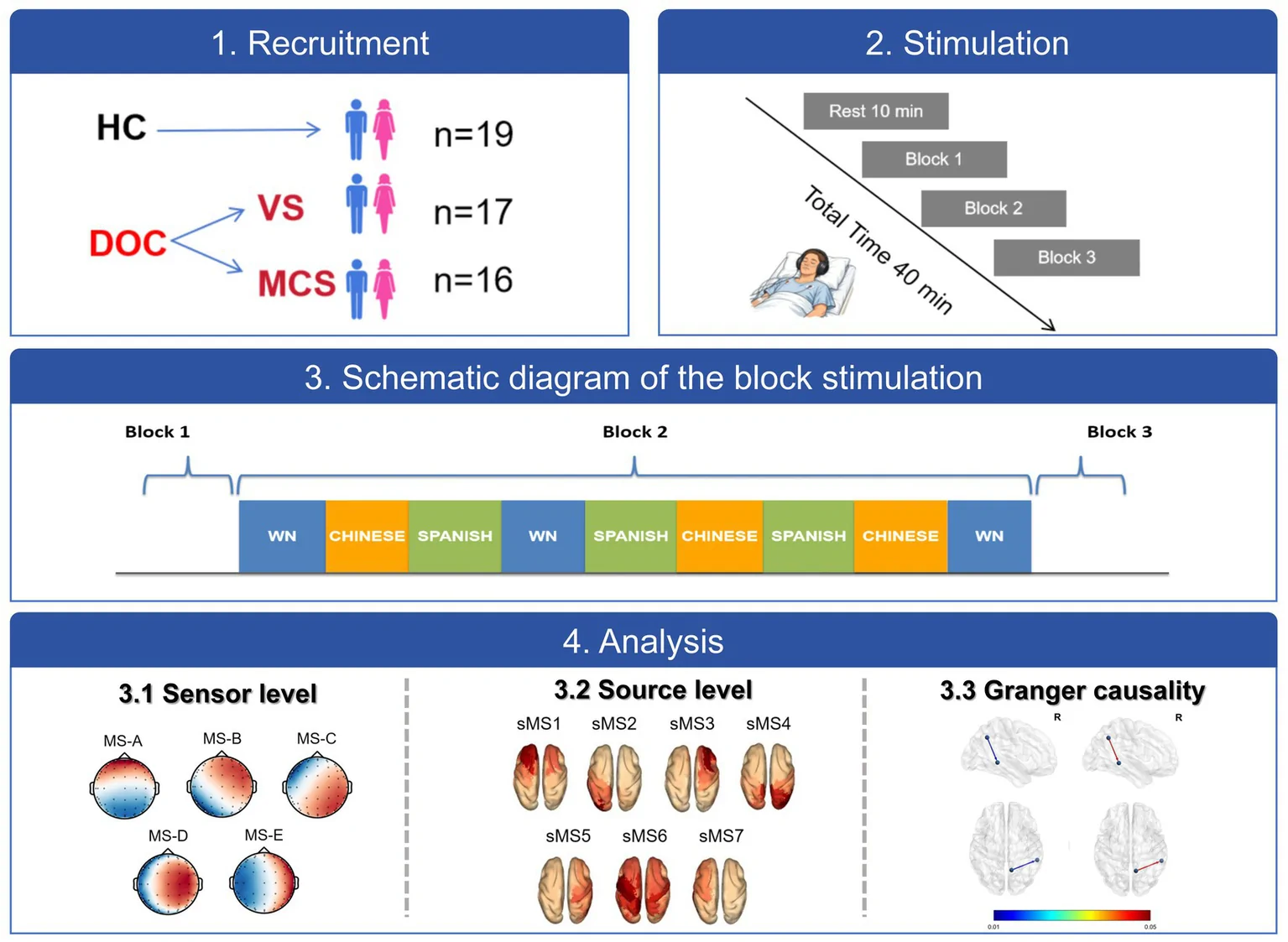
Schematic of the study protocol. **(1)** Participant recruitment; **(2)** experimental design; **(3)** stimulus procedure schematic; **(4)** microstate and functional connectivity analysis. WN, white noise.

Spanish segments, and three white noise segments. Within each block, the nine segments (Spanish, Chinese, white noise) were presented in random order. The two speech conditions were retained as separate conditions during acquisition but were collapsed for all inferential analyses (see Statistical Analysis).

### 2.3 EEG acquisition

EEG recordings were conducted in a quiet environment. Auditory stimuli were presented binaurally through wireless split-type earphones, with sound-insulating earmuffs (3 M PELTOR X5A, SNR 37 dB) worn to reduce ambient noise. A 10-min resting-state EEG was recorded as baseline, followed by an auditory stimulation phase of approximately 30 min. Participants were monitored to ensure wakefulness. Segments with severe artifacts (e.g., coughing, body movements) were marked during acquisition and excluded during offline preprocessing.

EEG data were acquired using the Neusen W64 wireless system (Neuracle). A total of 59 scalp channels were recorded according to the International 10–10 system (e.g., Fpz, Fz, Cz, Oz), with CPz as the online reference and a sampling rate of 1,000 Hz.

### 2.4 Data processing

EEG preprocessing was performed using the EEGLAB toolbox in MATLAB. The raw signal was downsampled to 500 Hz, band-pass filtered (1–45 Hz, zero-phase FIR), and notch filtered (48–52 Hz). Artifact segments were rejected through visual inspection, and bad channels were interpolated using spherical splines. Data were then re-referenced to the common average. Independent component analysis (ICA; Infomax algorithm) was applied to remove ocular and muscular artifacts.

#### 2.4.1 Source reconstruction

Source-level analysis was performed using the FieldTrip toolbox. The forward solution was computed using a standard boundary element method (BEM) head model based on the Montreal Neurological Institute (MNI) template. Electrode positions were aligned with the standard 10–10 electrode template, and a three-dimensional source grid with a spatial resolution of 10 mm was constructed within the brain volume. Anatomical parcellation was performed using the Automated Anatomical Labeling (AAL) atlas, retaining 78 cortical regions of interest (ROIs) for subsequent analysis (Oostenveld et al., 2011).

The inverse solution employed exact low-resolution electromagnetic tomography (eLORETA) to estimate source activity. According to the voxel-level linear formula of eLORETA, the current density estimate for the i-th voxel can be expressed as Equation (1):

$$y_{i}(n)=W_{i}^{-1}H_{i}^{T}{\left({\mathrm{HW}}^{-1}H^{T}+\mathrm{\alpha L}\right)}^{+}x(n)$$ (1)

where x(n) denotes the sensor-level EEG at time sample n, H_i_ is the lead-field matrix associated with voxel i, W_i_ is the corresponding weight submatrix, alpha is the regularization parameter, and (.)^^+^ denotes the Moore-Penrose pseudoinverse. The weight matrix is obtained through the iterative procedure in Equation (2):

$$W_{i}={\left[H_{i}^{T}{\left({\mathrm{HW}}^{-1}H^{T}+\mathrm{\alpha L}\right)}^{+}H_{i}\right]}^{1/2}$$ (2)

In the present implementation, the regularization parameter was set to 0.05. These expressions follow the standard eLORETA formulation in the source localization literature (Pascual-Marqui et al., 2011; Noroozi et al., 2023).

For each ROI, all valid source points were identified after atlas interpolation. When the source moment at a given grid point was three-dimensional, its Euclidean norm was first computed to obtain a scalar source amplitude. The representative ROI time series was then defined as the root-mean-square (RMS) across all valid source points within that ROI at each time sample. This approach was adopted to reduce signal cancelation caused by polarity differences between neighboring source estimates.

#### 2.4.2 Microstate analysis

Preprocessed sensor-level EEG and source-reconstructed ROI time series were both analyzed using the +microstate toolbox (Tait and Zhang, 2022). Sensor-level analysis retained five canonical microstate classes, and source-level analysis retained seven classes. Following standard microstate procedures, global field power (GFP) was computed at each time sample using Equation (3). A common cohort-level template set was used for individual back-fitting to retain comparable state definitions across participants and conditions (Tait and Zhang, 2022; Michel and Koenig, 2018). This choice is consistent with evidence supporting grand-mean fitting in sensor-level microstate analysis (Kleinert et al., 2024), but does not establish the reliability of the present source-space templates.

$$\mathrm{GFP}(t)=\sqrt{\frac{1}{N_{e}}\sum_{c=1}^{N_{e}}v_{c}{\left(t\right)}^{2}}$$ (3)

where $v_{c}$ denotes the average-referenced potential at electrode c, and $N_{e}$ is the number of electrodes. Microstate templates were estimated from EEG topographies sampled at local GFP maxima, where the signal-to-noise ratio is expected to be highest. During back-fitting, each time point was assigned to the microstate map displaying the maximum spatial correlation with the observed topography. Three conventional temporal parameters were extracted for each microstate: mean duration (ms), occurrence rate (times/s), and coverage (%). Duration reflects the stability of the neural network corresponding to the microstate; occurrence rate reflects the average number of activations of that network per second; and coverage reflects the proportion of total recording time during which that network is active (Tait and Zhang, 2022; von Wegner et al., 2018).

#### 2.4.3 Functional connectivity analysis

To investigate directed information flow between cortical regions involved in auditory processing and conscious awareness, we performed Granger causality (GC) analysis on the six cortical regions contributing most to source microstates 3 and 4, yielding 30 prespecified directed connections for group comparison. MVAR model adequacy was verified by confirming model stability (all eigenvalues of the coefficient matrix lying within the unit circle) and residual whiteness (Ljung–Box portmanteau test, *p* > 0.05). The representative source time series of these nodes were arranged as multivariate signal vectors and modeled as a multivariate autoregressive (MVAR) process. In the present analysis pipeline, the time series were divided into non-overlapping 12-s windows, and the MVAR model order was fixed at *p* = 10.

In the frequency domain, the transfer matrix and spectral density matrix are defined by Equations (4) and (5), respectively:

$$H(\omega )=A^{-1}(\omega )$$ (4)

$$S(\omega )=X(\omega )X^{†}(\omega )=H(\omega )\Sigma (\omega )H^{†}(\omega )$$ (5)

where A(ω) denotes the Fourier transform of the MVAR model coefficient matrix, Σ(ω) is the noise covariance matrix, and the superscript † denotes the conjugate transpose.

The frequency-domain Granger causality from process 2 to process 1 is computed as using Equation (6):

$$I_{2\to 1}(\omega )=\log\frac{S_{11}(\omega )}{{\bar{H}}_{11}(\omega )\Sigma_{11}{\bar{H}}_{11}^{*}(\omega )}$$ (6)

where S11(ω) is the autospectrum of process 1, Σ11 is the variance of the residual noise of process 1, and H11(ω) is the element of the modified transfer function.

Broadband GC is obtained by averaging the spectral GC over the analysis frequency range, as expressed by Equation (7):

$$F_{i\to j}=\frac{1}{\omega_{f}-\omega_{0}}\int_{\omega_{0}}^{\omega_{f}}I_{i\to j}(\omega )\;\mathrm{d\omega}$$ (7)

where ω_0_ and ωf denote the lower and upper limits of the analysis frequency band (e.g., 1–40 Hz), respectively.

The final broadband GC values were obtained by averaging the GC spectra across the full analysis frequency range within each time window, followed by averaging across windows. This procedure generated stable directed connectivity matrices for each auditory condition (rest, noise, and speech) (Bressler and Seth, 2011).

### 2.5 Statistical analysis

This study employed a Type III mixed-design analysis of variance (ANOVA), with group (HC, VS/UWS, MCS) as the between-subjects factor and auditory condition as the within-subject repeated-measures factor. Because the present hypotheses targeted contrasts among rest, acoustic stimulation, and speech rather than linguistic content per se, the two speech conditions were collapsed into a single speech condition by averaging condition-specific metrics within each participant, yielding three within-subject levels (resting state, white noise, speech). For each microstate class and metric (duration, occurrence rate, coverage), the main effects of group, the main effects of condition, and group × condition interaction effects were tested; *post hoc* estimated marginal-mean pairwise comparisons were corrected for multiple comparisons using the Holm method separately for the three group contrasts and the three condition contrasts within each class-by-metric family. Omnibus microstate tests were not additionally adjusted across classes or metrics. Group comparisons of Granger causality across the 30 directed connections were corrected for multiple comparisons using the Benjamini-Hochberg false discovery rate procedure; edge-wise uncorrected results were considered exploratory and are reported with exact *p*-values and effect sizes.

Sphericity was assessed using Mauchly’s test. Greenhouse–Geisser (GG)-corrected degrees of freedom and *p*-values were reported for all Condition and Group × Condition effects; GG correction was not applicable to the between-subject Group effect. Complete F statistics, degrees of freedom, and partial η^2^ are provided in Supplementary Tables S13, S14, and Mauchly results and GG epsilon in Supplementary Table S15. Significant interactions were followed by within-group simple Condition effects (GG-corrected) and estimated marginal-mean contrasts, with Holm correction across the three condition comparisons within each class-by-metric-by-group family. Comparisons without a significant corresponding omnibus effect were regarded as exploratory (Supplementary Tables S1–S9).

Etiology and sex were compared between MCS and VS/UWS using two-sided Fisher exact tests. Time since injury was compared using a Wilcoxon rank-sum test with continuity correction, and age using a Welch *t*-test. These comparisons describe baseline clinical characteristics and do not constitute covariate adjustment of the EEG analyses (Supplementary Table S12).

Receiver operating characteristic (ROC) analysis was performed to evaluate the individual-level discriminative performance of source-level microstate markers in two settings: HC versus DoC (19 HC, 33 DoC) and MCS versus VS/UWS (16 MCS, 17 VS/UWS). Candidate predictors comprised the coverage, occurrence rate, and mean duration of source microstates 3 and 4, averaged across auditory conditions to obtain condition-averaged markers. MS3 and MS4 were selected on the basis of group differences in this cohort before cross-validation.

Discrimination was evaluated using participant-level repeated stratified five-fold cross-validation with 100 repetitions. Missing-value imputation, standardization, single-marker score orientation, and multivariable model tuning (L2-regularized logistic regression and shrinkage linear discriminant analysis) were performed exclusively within the training folds. ROC curves were constructed from each participant’s out-of-fold probability averaged across repetitions, and 95% confidence intervals were obtained using stratified participant-level bootstrap resampling. Microstate template construction and the selection of MS3/MS4 preceded cross-validation and were not repeated within the outer training folds. The resulting estimates therefore describe internal discrimination conditional on the selected representation and features, rather than validation of the complete biomarker-discovery pipeline, residual optimism cannot be excluded.

## 3 Results

The MCS and VS/UWS groups did not differ significantly in etiology (*p* = 1.000), sex (*p* = 0.491), time since injury (*p* = 0.550), or age (*p* = 0.401) (Supplementary Table S12).

### 3.1 Sensor-level microstate performance of HC and DoC

To systematically evaluate group differences in sensor-level microstate metrics and their sensitivity to auditory conditions, we performed Type III mixed-design (group × condition) ANOVAs for each microstate class on duration, occurrence, and coverage. Group main effects were evaluated across conditions, and Condition main effects across groups, within the repeated-measures model. Holm-adjusted pairwise comparisons among the three groups are provided in Supplementary Table S1, condition main effects in Supplementary Table S2, condition pairwise comparisons in Supplementary Table S3, and Group × Condition interactions with follow-up simple-effect analyses in Supplementary Tables S7–S9. Complete sensor-level omnibus statistics, including partial η^2^, are reported in Supplementary Table S13; sphericity diagnostics are provided in Supplementary Table S15.

#### 3.1.1 Sensor-level EEG microstate topographies

Modified k-means clustering identified five prototypical microstate classes (microstates A–E) from the preprocessed 59-channel EEG data. Clustering and subsequent template matching ignored polarity; therefore, positive and negative reversals of the same spatial configuration were classified into the same microstate class (Pascual-Marqui et al., 2011; Michel and Koenig, 2018). The microstate maps obtained in this study were then registered and ordered against previously reported canonical EEG microstate templates A–D and their extended versions using spatial correlation/global map fitting (Michel and Koenig, 2018; Figure 2A).

**Figure 2:**
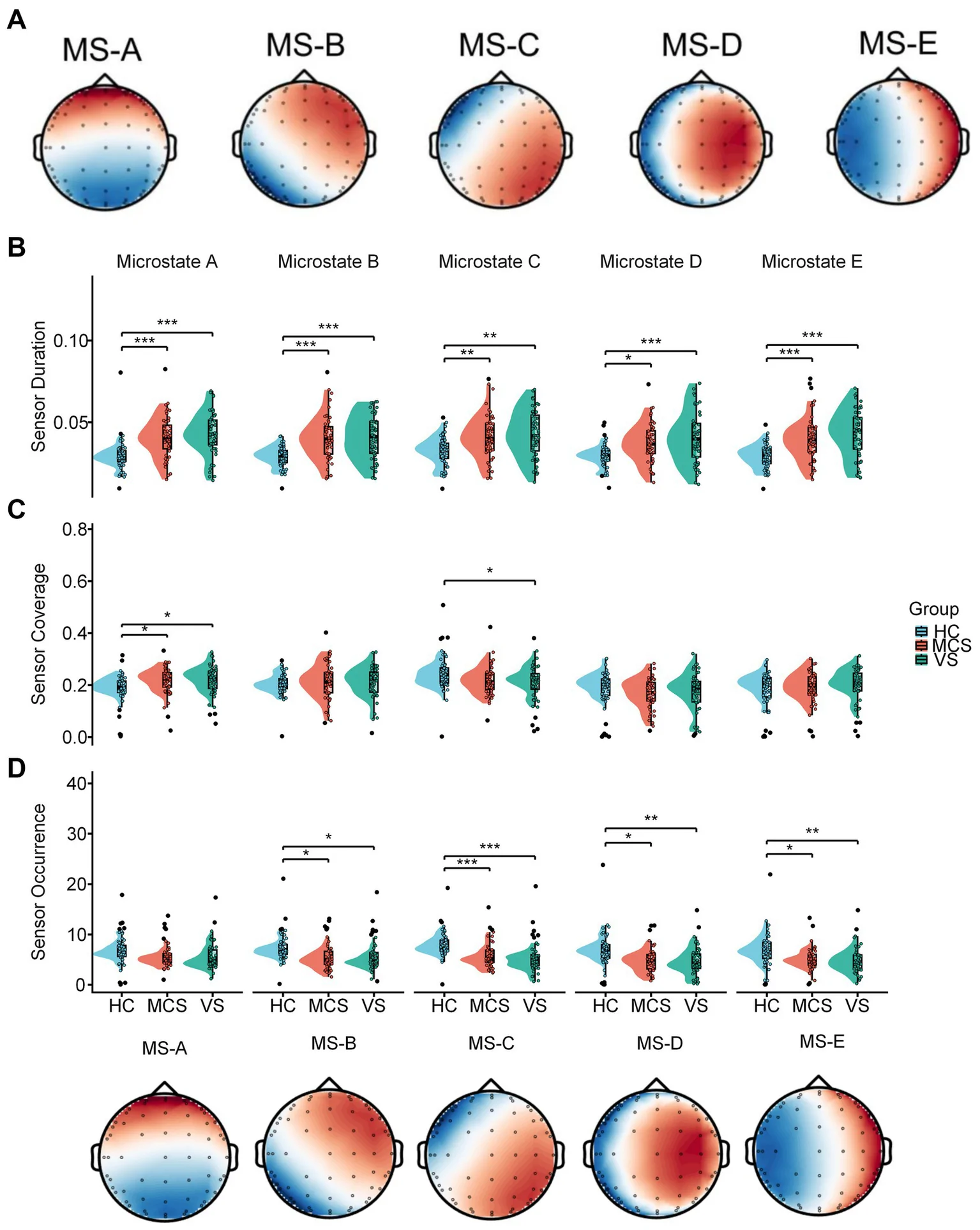
Sensor-level EEG microstate maps and metrics. **(A)** Sensor-level microstate maps (MS-A to MS-E), spatially ordered using a global map fitting algorithm and matched against canonical EEG microstate templates. **(B–D)** Sensor-level duration in seconds **(B)**, coverage as a proportion **(C)**, and occurrence rate per second **(D)** comparisons among healthy controls (HC), minimally conscious state (MCS), and vegetative state/unresponsive wakefulness syndrome (VS/UWS), with all conditions collapsed. Raincloud plots combine half-violin plots and box plots; individual data points are shown as dots. Significance symbols refer to Holm-adjusted group comparisons within each class-by-metric family (Supplementary Table S1): *p < 0.05, **p < 0.01, ***p < 0.001, ****p < 0.0001.

Consistent with prior reports, sensor-level EEG microstate topographies across all participants could be classified into five classes: MS-A exhibited high-potential regions concentrated over the frontal and anterior scalp, with low-potential regions distributed primarily over posterior and occipital areas, forming an anterior-to-posterior potential gradient that manifested as a dipole-like pattern from front to back. MS-B displayed a right-lateralized spatial pattern, with high-potential regions located mainly in the right frontotemporal area and low-potential regions in the left posterior scalp, producing a spatial reversal from right anterior to left posterior. MS-C also showed a lateralized oblique distribution, but its low-potential region was located in the left anterior area, while the high-potential region extended from the right temporoparietal area toward the right posterior scalp, forming a left-anterior-to-right-posterior gradient. MS-D exhibited pronounced left–right lateralization, with high-potential regions concentrated in the right central-temporal area and low-potential regions distributed over the left frontoparietal and posterior regions, displaying a predominantly interhemispheric asymmetry configuration. MS-E showed a more extensive bilateral potential distribution, with the low-potential region covering most of the left hemisphere and the high-potential region concentrated on the right lateral scalp, forming a stable left-negative/right-positive lateralization pattern. Because polarity was ignored during clustering, the red–blue directions described above may be globally inverted; what matters is the relative spatial configuration of each microstate.

These five microstate topographies remained qualitatively similar in spatial configuration across the healthy control (HC), vegetative state/unresponsive wakefulness syndrome (VS/UWS), and minimally conscious state (MCS) groups, with no obvious qualitative topographic distortions observed.

#### 3.1.2 Sensor-level microstate metrics

At the sensor level, the microstate model captured robust, interpretable dynamics along both experimental dimensions. Across groups, duration—the mean time a microstate remains dominant, reflecting stability—was the most sensitive metric: group main effects were significant for all five classes (all *p* < 0.001) (Supplementary Table S10), with HC showing shorter duration than both patient groups (Figure 2B and Supplementary Table S1) and no difference between MCS and VS/UWS (Supplementary Table S11). Occurrence, reflecting activation frequency, was likewise group-sensitive for microstates B–E (all *p* ≤ 0.05) (Supplementary Table S10; Figure 2D), with HC higher than both patient groups, but not for microstate A (Supplementary Table S10). Coverage was the least discriminatory, with group effects only for microstates A (*p* = 0.007) and C (*p* = 0.021) (Supplementary Table S10; Figure 2C). Across conditions, the model was equally responsive: duration and occurrence showed significant condition main effects (all GG-corrected *p* < 0.001) (Supplementary Table S2), with shorter duration and higher occurrence during rest than during noise and speech (Supplementary Table S3; Supplementary Figures S1A, S1C), whereas coverage did not vary across conditions (Supplementary Table S2; Supplementary Figure S1B). These condition effects were partly group-dependent—significant Group × Condition interactions emerged for class E occurrence, class A coverage, and class E coverage (all GG-corrected *p* ≤ 0.015) (Supplementary Tables S7, S8), with simple effects strongest in HC; class E occurrence also varied across conditions in MCS, and class E coverage in VS/UWS (Supplementary Tables S8, S9)—indicating that auditory modulation of microstate dynamics is itself altered in DoC.

Taken together, these results validate the sensor-level microstate model on both dimensions: it cleanly separated healthy controls from DoC patients and resolved the three auditory conditions. However, its discriminatory power did not extend to the clinically decisive distinction—no sensor-level metric differentiated MCS from VS/UWS. This limitation motivated the source-level analysis reported below.

### 3.2 Source-level microstate performance of HC and DoC

#### 3.2.1 Source-level EEG microstate topographies

In the sensor-level analysis above, we found that sensor-level microstate analysis could effectively distinguish HC from DoC patients but was unable to differentiate VS/UWS from MCS. To further mine microstate metrics capable of distinguishing VS/UWS from MCS, we performed source localization on high-density EEG data. After projecting EEG signals onto cortical source space via eLORETA inverse solution, k-means clustering (*k* = 7) identified seven source-level microstate classes (microstates 1–7) (Zhang et al., 2023). Figure 3 displays the current density distributions for each microstate on the standard cortical surface (warm colors indicate regions of relatively high current density). The spatial characteristics of each microstate are as follows:

**Figure 3:**
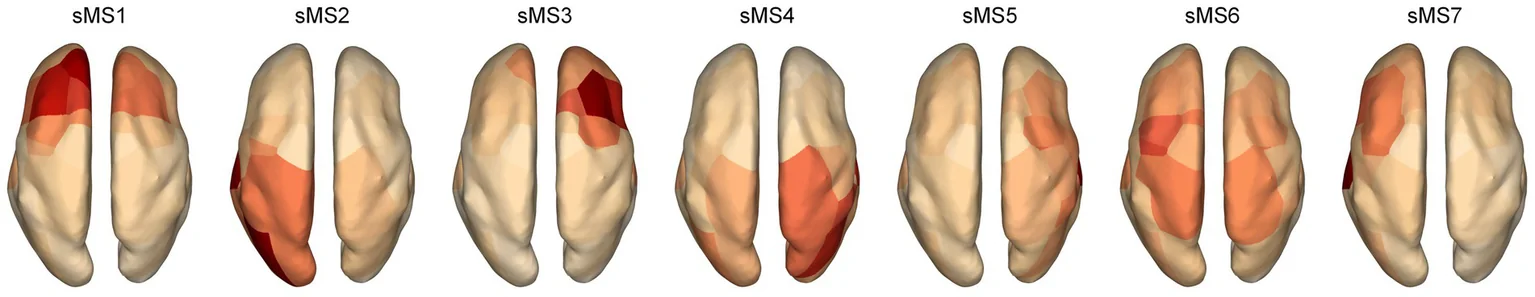
Source-reconstructed EEG microstate maps. Source-reconstructed microstate maps were generated by k-means clustering (*k* = 7).

MS1: High current density was distributed mainly over the bilateral anterior-superior-lateral cerebral cortex, dominated by a bilateral anterior-superior pattern extending from the frontal lobe toward the superior parietal lobule, with relatively weak posterior activation. MS2: Current density was concentrated in the bilateral posterior occipital lobe (posterior pole), with clear synchronous activation visible at both occipital poles, exhibiting a typical bilateral symmetric posterior distribution with limited anterior cortical involvement. MS3: Current density showed a marked right frontal unilateral dominance. A large area of high-density activation (deep red) was visible on the right frontal convexity, encompassing the middle frontal gyrus, inferior frontal gyrus, and superior frontal gyrus (corresponding to the dorsolateral prefrontal, ventrolateral prefrontal, and orbitofrontal subregions, respectively); activation in the homologous left frontal region was markedly weaker. This spatial configuration suggests dominant engagement of the right prefrontal cognitive control network. MS4: Current density was preferentially distributed over the bilateral posterior-medial cerebral hemisphere, concentrated at the parieto-occipital junction and extending posteriorly into the temporal lobe, with particularly prominent activation in the right hemisphere. The strongest contributing nodes included the right precuneus, right middle temporal gyrus, and angular gyrus—the spatial topography of these regions overlaps extensively with the default mode network (DMN) and the posterior cortical hot zone. MS5: Current density exhibited a focal unilateral distribution, with activation confined mainly to the lateral surface of one hemisphere; its spatial extent was markedly more restricted than MS3, failing to form the widespread frontal or posterior network patterns seen in the other classes.

Source-level microstate duration and occurrence showed significant group effects for nearly all classes, whereas coverage effects were largely confined to MS3 and MS4 (Table 2). Only MS3 and MS4 further differentiated VS/UWS from MCS (Table 2), as detailed below:

**Table 2:** Source-level microstate metrics and group comparisons.

(A) Duration, occurrence, and coverage of source-level microstates for HC, VS/UWS, and MCS patients; the last column reports *F*-values (*P*-values)					
Variable	MS	HC M(SE)	MCS M(SE)	VS M(SE)	F(*p*)
Duration	1	24.895 (1.372)	34.552 (2.698)	34.921 (3.476)	5.05 (0.010*)
	2	21.663 (1.043)	34.278 (3.788)	35.381 (2.890)	8.474 (<0.001***)
	3	21.286 (0.694)	32.354 (2.530)	42.134 (4.937)	11.519 (<0.001***)
	4	22.394 (1.103)	40.291 (5.103)	32.697 (1.974)	8.987 (<0.001***)
	5	22.362 (0.962)	30.3 (1.129)	32.894 (2.932)	9.041 (<0.001***)
	6	25.528 (1.565)	36.271 (2.682)	31.221 (2.156)	6.46 (0.003**)
	7	21.163 (0.644)	31.589 (2.147)	35.525 (3.155)	12.372 (<0.001***)
Occurrence rate	1	7.617 (0.678)	4.802 (0.533)	4.261 (0.474)	10.083 (<0.001***)
	2	4.333 (0.505)	2.026 (0.387)	2.618 (0.605)	5.636 (0.006**)
	3	5.071 (0.573)	2.835 (0.644)	5.43 (0.539)	5.498 (0.007**)
	4	3.658 (0.505)	3.028 (0.646)	0.845 (0.237)	9.235 (<0.001***)
	5	6.742 (0.669)	3.465 (0.477)	4.366 (0.501)	9.052 (<0.001***)
	6	8.737 (0.622)	5.625 (0.771)	4.522 (0.678)	10.615 (<0.001***)
	7	5.474 (0.656)	3.726 (0.546)	4.638 (0.869)	1.533 (0.226)
Coverage	1	19.721 (2.316)	18.119 (2.682)	15.224 (2.356)	0.891 (0.417)
	2	9.836 (1.442)	8.441 (3.132)	9.205 (2.531)	0.086 (0.918)
	3	10.916 (1.328)	11.096 (3.506)	25.172 (4.128)	6.88 (0.002**)
	4	8.801 (1.510)	15.338 (4.648)	3.356 (1.447)	4.443 (0.017*)
	5	15.591 (1.752)	11.467 (1.777)	14.893 (2.130)	1.32 (0.276)
	6	23.425 (2.840)	22.677 (4.027)	14.966 (2.668)	2.165 (0.126)
	7	11.711 (1.476)	12.862 (2.729)	17.183 (3.989)	1.041 (0.361)

(B) *Post hoc* comparisons of source-level microstate metrics between VS/UWS and MCS patients; the last column reports t ratios (Holm-corrected *P*-values); negative *t*-values indicate MCS < VS/UWS				
Variable	*MS*	*MCS M(SE)*	*VS M(SE)*	t (*p*)
Duration	1	34.552 (2.698)	34.921 (3.476)	−0.098 (0.922)
	2	34.278 (3.788)	35.381 (2.890)	−0.284 (0.777)
	3	32.354 (2.530)	42.134 (4.937)	−2.154 (0.036*)
	4	40.291 (5.103)	32.697 (1.974)	1.737 (0.089†)
	5	30.3 (1.129)	32.894 (2.932)	−0.958 (0.343)
	6	36.271 (2.682)	31.221 (2.156)	1.64 (0.119)
	7	31.589 (2.147)	35.525 (3.155)	−1.257 (0.215)
Occurrence rate	1	4.802 (0.533)	4.261 (0.474)	0.643 (0.523)
	2	2.026 (0.387)	2.618 (0.605)	−0.797 (0.429)
	3	2.835 (0.644)	5.43 (0.539)	−3.058 (0.011*)
	4	3.028 (0.646)	0.845 (0.237)	3.081 (0.007**)
	5	3.465 (0.477)	4.366 (0.501)	−1.09 (0.281)
	6	5.625 (0.771)	4.522 (0.678)	1.108 (0.273)
	7	3.726 (0.546)	4.638 (0.869)	−0.89 (0.756)
Coverage	1	18.119 (2.682)	15.224 (2.356)	0.817 (0.836)
	2	8.441 (3.132)	9.205 (2.531)	−0.222 (1.000)
	3	11.096 (3.506)	25.172 (4.128)	−3.126 (0.006**)
	4	15.338 (4.648)	3.356 (1.447)	2.979 (0.013*)
	5	11.467 (1.777)	14.893 (2.130)	−1.249 (0.436)
	6	22.677 (4.027)	14.966 (2.668)	1.672 (0.202)
	7	12.862 (2.729)	17.183 (3.989)	−1.048 (0.599)

(C) Summary of temporal parameters for source microstates 3 and 4 across consciousness-level groups			
Parameter	MS3 (right prefrontal)	MS4 (right Parieto-Occipito-Temporal)	Direction of change
Coverage	VS/UWS > MCS	MCS > VS/UWS	Opposite
Duration	VS/UWS > MCS	MCS > VS/UWS†	Opposite
Occurrence rate	VS/UWS > MCS	MCS > VS/UWS	Opposite

M (SE) = mean (standard error). Duration is expressed in milliseconds, coverage as a percentage, and occurrence rate per second. Group F tests used degrees of freedom (2, 49), and *post-hoc t-*tests used 49 degrees of freedom. Partial η^2^ values are reported in Supplementary Table S14. Descriptive means and SEs were calculated from participant-level averages across conditions; these SEs differ from the model-based estimated marginal-mean SEs in Supplementary Table S4. *p < 0.05. **p < 0.01. ***p < 0.001. †0.05 ≤ *p* < 0.10.

#### 3.2.2 Source-level microstate coverage

Coverage refers to the proportion of total recording time during which a given microstate is dominant. Among source-level microstate coverage metrics, MS3 and MS4 showed the strongest discriminatory power between MCS and VS/UWS, with the two patient groups changing in opposite directions. Specifically, MS3 coverage differed significantly across groups (*p* = 0.002) (Table 2); *post-hoc* tests showed VS/UWS > MCS (*p* = 0.006) (Table 2; Figure 4A) and VS/UWS > HC (*p* = 0.005) (Supplementary Table S4), with no significant difference between HC and MCS (Figure 4A). MS4 coverage also differed significantly across groups (*p* = 0.017) (Table 2); *post-hoc* comparisons showed MCS > VS/UWS (*p* = 0.013) (Table 2; Figure 4A), with no significant differences between HC and either patient subgroup (Figure 4A). These findings indicate that MS3 and MS4 coverage exhibit diametrically opposite patterns in MCS versus VS/UWS.

**Figure 4:**
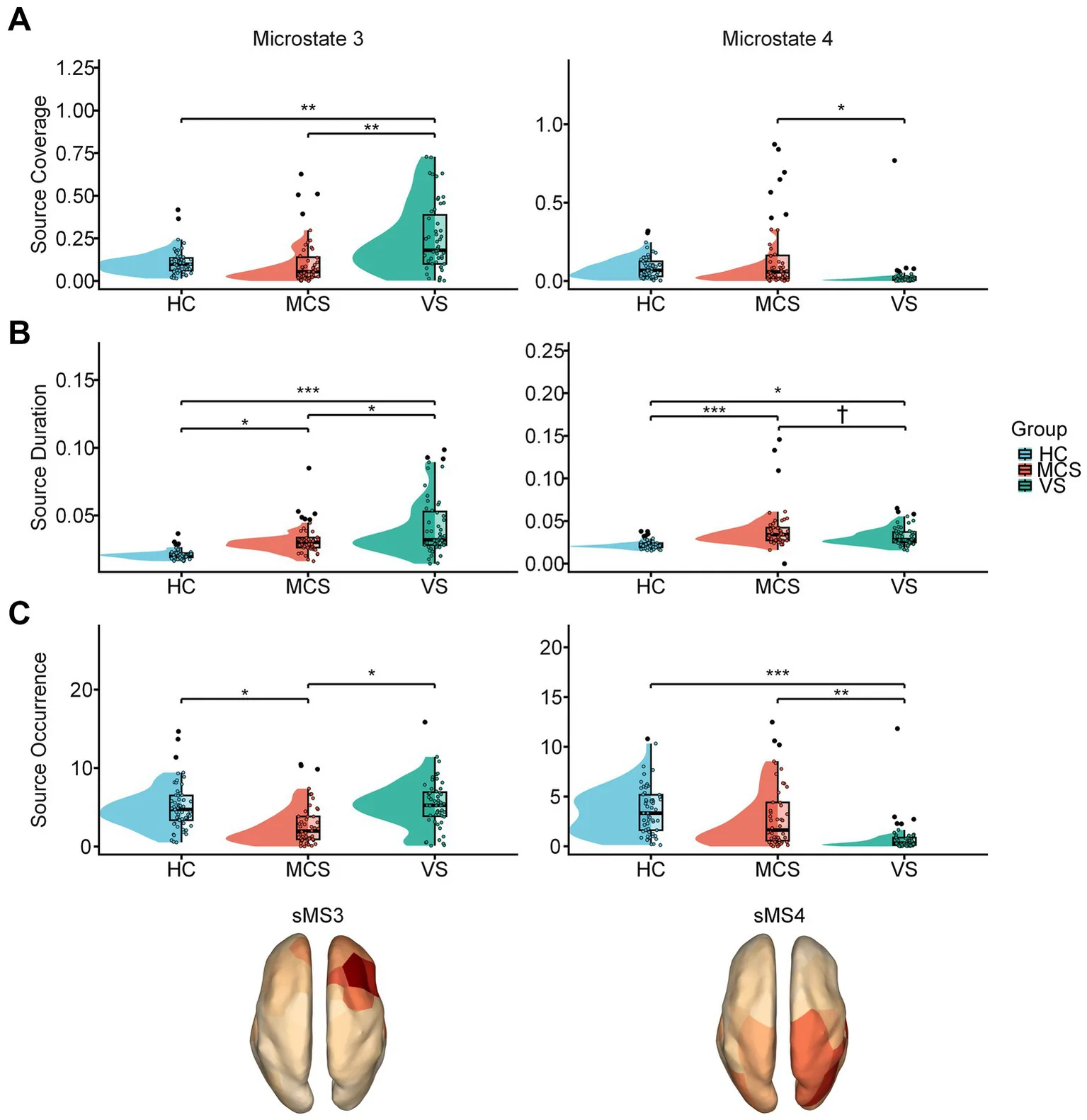
Source-level microstate metrics for source microstates 3 and 4 across HC, MCS, and VS/UWS groups. sMS3 and sMS4 denote source microstates 3 and 4, respectively. Each panel shows source coverage, source duration, and source occurrence for sMS3 and sMS4, with the corresponding source-space maps at the bottom. **(A)** coverage as a proportion, **(B)** duration in seconds, **(C)** occurrence rate per second; left column: sMS3, right column: sMS4. Raincloud plots combine half-violin plots, box plots, and individual data points. Significance symbols refer to Holm-adjusted group comparisons within each class-by-metric family (Supplementary Table S4): *p < 0.05, **p < 0.01, ***p < 0.001, ****p < 0.0001. †0.05 ≤ *p* < 0.10. Duration is plotted in seconds and coverage as a proportion; Table 2 reports duration in milliseconds and coverage as a percentage. Cyan, coral, and green denote HC, MCS, and VS/UWS, respectively.

#### 3.2.3 Source-level microstate duration

Duration refers to the mean length of time (in milliseconds) that a microstate remains dominant before transitioning to the next state. Source-level microstate duration showed significant group differences across multiple classes. MS3 duration differed significantly across groups (*p* < 0.001) (Table 2); *post-hoc* tests showed VS/UWS > MCS (*p* = 0.036) (Table 2; Figure 4B), HC shorter than MCS (*p* = 0.031) (Supplementary Table S4), and HC shorter than VS/UWS (*p* < 0.001). MS4 duration also showed significant differences (*p* < 0.001) (Table 2); both MCS and VS/UWS were longer than HC (MCS vs. HC: *p* < 0.001; VS/UWS vs. HC: *p* = 0.035), whereas the trend for MCS > VS/UWS did not reach significance (*p* = 0.089) (Table 2; Figure 4B). Thus, MS3 duration was elevated in VS/UWS, whereas MS4 duration tended to be relatively higher in MCS.

#### 3.2.4 Source-level microstate occurrence

Occurrence refers to the average number of times per second that a given microstate appears. Source-level microstate occurrence also indicated that MS3 and MS4 could distinguish MCS from VS/UWS, with group differences in opposite directions. MS3 occurrence differed significantly across groups (*p* = 0.007) (Table 2); *post-hoc* comparisons showed VS/UWS > MCS (*p* = 0.011) (Table 2; Figure 4C) and HC > MCS (*p* = 0.019) (Supplementary Table S4), with no difference between HC and VS/UWS. MS4 occurrence also showed significant differences (*p* < 0.001) (Table 2); *post-hoc* tests showed MCS > VS/UWS (*p* = 0.007) (Table 2; Figure 4C) and HC > VS/UWS (*p* < 0.001), with no significant difference between HC and MCS (Figure 4C). In summary, MS3 was enhanced in VS/UWS whereas MS4 was enhanced in MCS, suggesting that these two microstates capture opposing patterns of source-space microstate dynamics under different conscious states. At the source level, no significant GG-corrected Condition main effects or Group × Condition interactions were detected for any microstate metric (Supplementary Tables S5, S14); condition pairwise comparisons at the source level are reported in Supplementary Table S6.

### 3.3 Core source-level biomarkers of consciousness level: MS3 and MS4

Among the seven source-level microstates, MS3 differed significantly between VS/UWS and MCS across all three temporal parameters, and MS4 differed significantly for coverage and occurrence, with duration showing the same directional pattern at a marginal level (*p* = 0.089) (Supplementary Table S4; Figure 4); no other microstate class showed consistent group differences, identifying MS3 and MS4 as the core spatial biomarkers for discriminating consciousness level. Notably, MS3 and MS4 showed diametrically opposite directional patterns: MS3 parameters were elevated in VS/UWS, whereas MS4 parameters were elevated in MCS. The remaining source-level microstates (MS1, MS2, MS5, MS6, MS7) did not show consistent significant differences across groups, suggesting that these microstates may reflect basic EEG fluctuation patterns shared by patients at different consciousness levels rather than specific neural markers that distinguish VS/UWS from MCS.

### 3.4 ROC analysis

Individual-level discrimination of consciousness status was evaluated using participant-level repeated stratified five-fold cross-validation with 100 repetitions. Missing-value imputation, standardization, single-marker score orientation, and multivariable model tuning were performed exclusively within the training folds. ROC curves were constructed from each participant’s out-of-fold probability averaged across repetitions, and 95% confidence intervals for AUC were obtained using stratified participant-level bootstrap resampling. Template construction and MS3/MS4 selection preceded cross-validation, so these estimates represent internal evaluation conditional on those upstream choices.

#### 3.4.1 Discrimination between HC and DoC

The HC-versus-DoC analysis included 52 participants: 19 HC participants and 33 patients with DoC (16 MCS and 17 VS/UWS). The L2-regularized logistic model combining source-level MS3 and MS4 duration, coverage, and occurrence achieved the highest internally validated discrimination (AUC = 0.982, 95% bootstrap CI [0.949, 1.000]), with a mean sensitivity of 0.864 and a mean specificity of 0.928. Because the healthy control group was younger than the patient groups and validation was internal to this cohort, this estimate should be interpreted as an upper bound pending external, demographically controlled validation. The six-marker shrinkage LDA model yielded an AUC of 0.962 [95% CI (0.907, 1.000)], a mean sensitivity of 0.870, and a mean specificity of 0.910. Among the single markers, MS3 duration and MS4 duration showed the strongest performance, with AUCs of 0.901 [95% CI (0.805, 0.976); sensitivity = 0.846, specificity = 0.849] and 0.896 [95% CI (0.788, 0.979); sensitivity = 0.918, specificity = 0.790], respectively. Thus, both duration markers strongly discriminated HC from DoC, while the combined six-marker model provided the highest internal cross-validated performance (Figures 5A,B).

**Figure 5:**
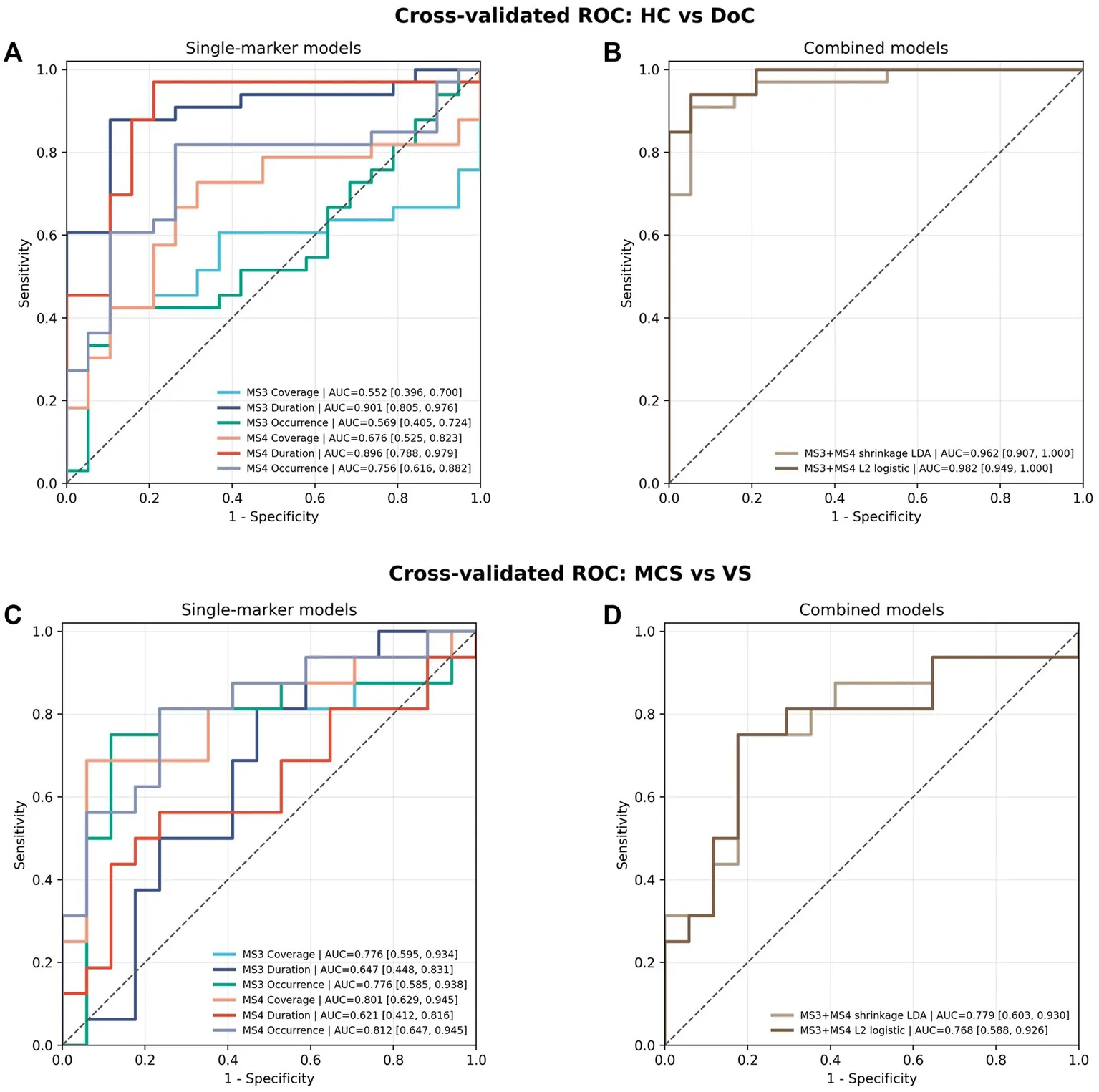
Cross-validated receiver operating characteristic (ROC) curves for source microstate 3 and 4 metrics. **(A,B)** Discrimination between HC and DoC: **(A)** single-marker models and **(B)** combined models. **(C,D)** Discrimination between MCS and VS/UWS: **(C)** single-marker models and **(D)** combined models. Curves display sensitivity as a function of 1 − specificity; AUC values with 95% bootstrap confidence intervals are reported in the legend. Template construction and MS3/MS4 selection were performed before cross-validation; the curves represent internal evaluation conditional on these choices.

#### 3.4.2 Discrimination between MCS and VS/UWS

The patient subgroup analysis included 16 patients with MCS and 17 with VS/UWS. MS4 occurrence yielded the highest cross-validated AUC [AUC = 0.812, 95% bootstrap CI (0.647, 0.945)], with a mean sensitivity of 0.679 and a mean specificity of 0.715. MS4 coverage showed comparable discrimination [AUC = 0.801, 95% CI (0.629, 0.945)], with a mean sensitivity of 0.619 and a mean specificity of 0.898. MS3 occurrence and MS3 coverage each yielded an AUC of 0.776, with 95% CIs of (0.585, 0.938) and (0.595, 0.934), respectively. In contrast, MS3 and MS4 duration yielded AUCs of 0.647 and 0.621. The six-marker shrinkage LDA and nested L2-regularized logistic models yielded AUCs of 0.779 (95% CI [0.603, 0.930]) and 0.768 (95% CI [0.588, 0.926]), respectively, and therefore did not outperform the single MS4 occurrence or coverage markers. These findings indicate that the strongest internally validated information for distinguishing MCS from VS/UWS was provided by MS4 occurrence and coverage. Because the patient sample was modest and the confidence intervals remained wide, independent external validation is required (Figures 5C,D).

### 3.5 Exploratory Granger causality analysis

In line with the prespecified analytic hierarchy (see Introduction), the following connectivity analysis is exploratory and hypothesis-generating; no diagnostic claims are derived from it. In the present implementation, frequency-domain Granger causality was computed using the standard FieldTrip pipeline. Broadband GC was obtained by averaging the GC spectrum across the analyzed frequency range within each time window and then averaging across windows. The exported GC table was direction-corrected so that the reported connection denotes the directional influence from the source ROI to the target ROI. As the noise and speech conditions showed no significant difference (see below), they were pooled into a single Auditory condition for the primary GC analysis. MCS and VS/UWS were then compared across the 30 directed connections formed by the six ROIs. No connection survived Benjamini–Hochberg FDR correction (all *q* > 0.05), indicating that the data did not support a multiplicity-corrected group difference at the six-ROI network level.

In the uncorrected edge-wise analysis, the right middle temporal gyrus-to-right precuneus connection (Temporal_Mid_R → Precuneus_R; T → P) yielded the smallest *p* value. Raw Auditory GC was 0.0456 ± 0.0612 in MCS (*n* = 16) and 0.0141 ± 0.0136 in VS/UWS (*n* = 17). Following log10(GC + 10^−6^) transformation, the corresponding group means were −1.650 ± 0.538 and −2.069 ± 0.490. Welch’s test indicated an uncorrected group difference, *t*(30.28) = 2.334, *p* = 0.026, Hedges’ *g* = 0.795; the log-scale mean difference was 0.419, 95% CI (0.053, 0.785). However, this edge did not remain significant after BH-FDR correction across the 30 directed edges, *q* = 0.452. A Mann–Whitney sensitivity analysis of the raw GC values yielded *U* = 194, *p* = 0.038. Accordingly, the T → P result should be interpreted as an uncorrected exploratory network signal rather than as the only connection surviving multiplicity correction (Figure 6B).

**Figure 6:**
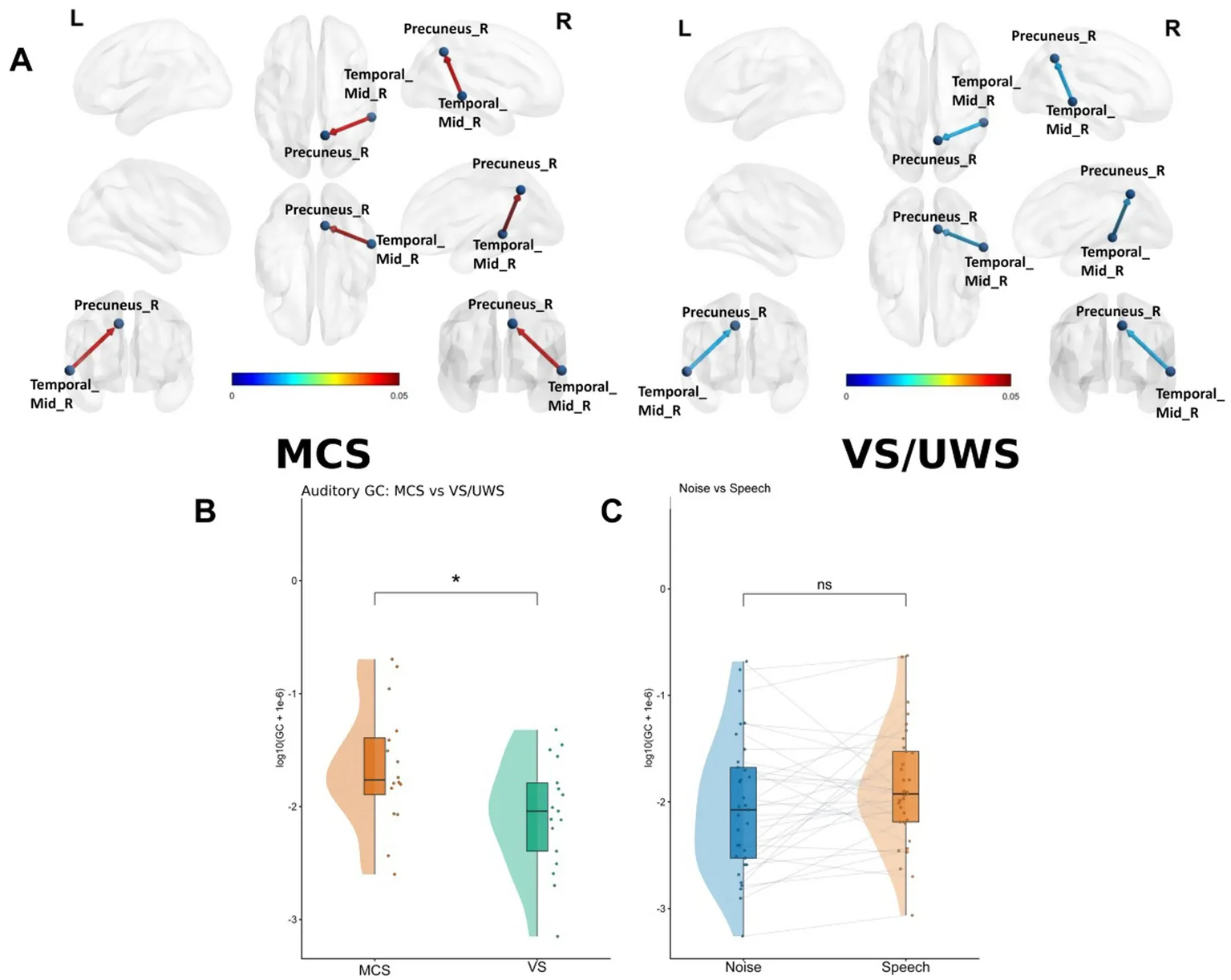
Source-space Granger causality results for the candidate temporal-to-precuneus pathway. **(A)** Anatomical visualization of the directed pathway from the right middle temporal gyrus (Temporal_Mid_R) to the right precuneus (Precuneus_R), projected onto transparent cortical surfaces from multiple perspectives for the MCS (left) and VS/UWS (right) groups; the color bar indicates GC strength. **(B)** Comparison of auditory GC values between MCS and VS/UWS. **(C)** Within-patient comparison of GC values between noise and speech conditions. Values are plotted as log10(GC + 1e−6). Raincloud plots combine half-violin plots, box plots, paired or individual data points, and statistical annotations. Statistical annotations are uncorrected: *p < 0.05; ns indicates not significant. No group difference survived BH-FDR correction across the 30 directed edges (see section 3.5).

Noise and Speech did not show a clear overall condition difference (paired log-scale test, *p* = 0.083; Wilcoxon signed-rank test, *p* = 0.183).

Overall, the results suggest potentially stronger directed information flow from the right middle temporal gyrus to the right precuneus in MCS, but this finding did not survive network-level correction and requires confirmation in an independent sample. The anatomical locations of the two connected regions are visualized in Figure 6A for the MCS (left) and VS/UWS (right) groups.

## 4 Discussion

By integrating source-level EEG microstate analysis with directed functional connectivity metrics from a multi-condition auditory paradigm, this study investigated key neural circuit markers underlying different levels of consciousness in patients with disorders of consciousness (DoC). Three main findings emerged. First, source-reconstructed microstates 3 (frontal-dominant) and 4 (parietal-occipital-temporal-dominant) displayed opposite patterns between VS/UWS and MCS patients. Second, in repeated internal cross-validation, MS4 occurrence discriminated MCS from VS/UWS with an AUC of 0.812 [95% bootstrap CI (0.647, 0.945)]; notably, combined multivariable models did not outperform this single marker, indicating that the discriminative information is concentrated in a parsimonious, deployment-friendly index. Third, among 30 directed connections, information flow from the right middle temporal gyrus to the right precuneus was nominally stronger in MCS than in VS/UWS (Welch’s *p* = 0.026, uncorrected; Hedges’ *g* = 0.795) but did not survive false discovery rate correction (*q* = 0.452) and should therefore be interpreted as preliminary, hypothesis-generating evidence. These findings converge on the principle that consciousness-level differences depend on the integrity of directed bottom-up sensory-to-integration pathways.

A methodological note is warranted on the combined use of source-resolved microstates and Granger causality, which are not competing feature-extraction techniques but operate at different levels of description. Microstates capture transient, whole-brain topographic configurations—which large-scale network state is dominant, how long it lasts, and how often it occurs—and are thus state-level, descriptive, and data-driven. Granger causality estimates directed functional influence between preselected regions and is thus model-based, directed, and circuit-level. Combining complementary EEG descriptors in this manner follows established practice in disorders-of-consciousness research. Sitt et al. (2014)systematically screened a comprehensive library of EEG markers—spanning event-related potentials, spectral power, signal complexity, and inter-electrode information exchange—across 181 recordings and showed that measures from different descriptive families synergize when combined; Engemann et al. (2018)extended this multi-feature framework to robust, cross-site and cross-protocol classification of consciousness states; Chennu et al. (2017)showed that EEG-derived network measures integrate with PET metabolism to predict diagnosis and prognosis at the bedside; and Gui et al. (2020) jointly analyzed speech-tracking responses and global microstate dynamics, concluding that “combining different analytical methods could deliver better diagnostic and predictive capabilities.” This practice is further endorsed at the guideline level: the European Academy of Neurology recommends that standardized clinical evaluation, EEG-based techniques, and functional neuroimaging be integrated for the multimodal evaluation of DoC, with the state of consciousness classified according to the highest level revealed by any of these approaches (De Herdt, 2020). In the present study, however, the two methods were assigned deliberately asymmetric roles. All diagnostic claims rest on the microstate analysis alone; consistently with this parsimonious design, the combined multivariable models did not outperform the single MS4 occurrence marker, and no diagnostic value is claimed for the connectivity metrics. The Granger causality analysis was prespecified solely as a hypothesis-generating probe of circuit mechanisms, with its own conservative multiplicity control (30 prespecified directed edges, Benjamini–Hochberg FDR), and its network-level null result is reported in full. The primary conclusions of this study therefore do not depend on the connectivity analysis.

Mapped back onto the two *a priori* hypotheses, the results give a clear and asymmetric verdict. Hypothesis 1 was supported: source-level microstates revealed dissociable, diametrically opposite prefrontal (MS3) and posterior (MS4) signatures that differed between VS/UWS and MCS in five of the six MS3/MS4 temporal-parameter contrasts; MS4 duration did not reach significance (Holm-adjusted *p* = 0.089) (Supplementary Table S4). Hypothesis 2, as prespecified across the full set of 30 directed connections, was not supported after false-discovery-rate correction: no directed connection showed a multiplicity-corrected group difference, and only the right middle temporal-to-precuneus edge reached nominal, uncorrected significance. We regard this transparent null as a finding rather than a failure: it delimits what a cohort of this size can and cannot support, and it defines the specific circuit hypothesis that future, adequately powered studies should test.

### 4.1 Opposing prefrontal and posterior microstate dynamics

Source-localized microstate analysis revealed two opposing neurodynamic phenotypes: VS/UWS patients exhibited elevated MS3 (right prefrontal) parameters alongside attenuated MS4 (posterior parieto-occipito-temporal) parameters, whereas MCS patients showed the reverse pattern—consistent in direction across all three temporal metrics and significant in five of the six MS3/MS4 contrasts (Table 2; Figure 4). This bipolar opposition aligns with the theoretical prediction that consciousness depends on dynamic information exchange between prefrontal broadcasting and posterior integration networks (Mashour et al., 2020; Baars, 2005; Giacino et al., 2004). In VS/UWS, prefrontal predominance without effective posterior engagement may reflect a dissociated state in which arousal-related prefrontal signals fail to propagate to consciousness-generating posterior hubs; conversely, MCS patients retain the capacity for posterior sensory integration, reflected in enhanced MS4 parameters and preserved bottom-up temporal-to-precuneus connectivity. That the two phenotypes form a systematic seesaw rather than a unidirectional decline suggests that consciousness level indexes the balance between opponent prefrontal and posterior networks, rather than the integrity of either network alone.

### 4.2 Auditory modulation at sensor and source levels

Auditory condition modulated sensor-level microstate duration and occurrence but showed no main effect on any source-level metric. This dissociation is plausible because sensor topographies mix multiple cortical generators, whereas source reconstruction and aggregation into seven classes may reduce sensitivity to condition-related variance. The MS3/MS4 group differences may reflect features that are less sensitive to the auditory contexts examined here. However, the absence of detectable source-level condition effects does not establish stimulus independence, temporal stability, or equivalent discriminative performance across conditions. Throughout, null noise-versus-speech comparisons indicate an absence of detectable differences at the present sample size, not equivalence of conditions.

### 4.3 Exploratory temporal-to-precuneus connectivity

Across the 30 directed connections examined, one nominally significant effect emerged: information flow from the right middle temporal gyrus to the right precuneus was higher in MCS than in VS/UWS (Welch’s *p* = 0.026, uncorrected; Hedges’ *g* = 0.795). This effect did not survive Benjamini–Hochberg correction across the 30 edges, and a Mann–Whitney sensitivity analysis yielded a consistent uncorrected result; we therefore interpret this pathway as preliminary evidence for—rather than confirmation of—a bottom-up circuit whose integrity may differentiate consciousness levels (Figures 6A,B). Anatomically, the pathway may be supported by the posterior segment of the perisylvian arcuate fasciculus–superior longitudinal fasciculus complex, which connects the posterior middle temporal gyrus to the angular gyrus (Martino et al., 2013), with subsequent short association fibers to the precuneus, a core node of the default mode network and a putative consciousness hub (Dadario and Sughrue, 2023; Siclari et al., 2017; Leech and Sharp, 2014). The absence of statistically detectable condition differences (Figure 6C), if confirmed in larger samples, would be consistent with evidence that consciousness correlates with the capacity for long-distance network integration regardless of input modality (Rosanova et al., 2012; Casali et al., 2013; Casarotto et al., 2016). This candidate pathway also converges with recent graph-learning evidence from dual-modal MRI published in this journal, in which the middle temporal gyrus ranked among the most discriminative regions for consciousness-level classification (Qi et al., 2024).

### 4.4 Clinical implications and future directions

The EEG-based biomarker framework presented here offers three potential clinical advantages. First, the MS3/MS4 temporal parameters provide a quantitative index of consciousness level obtainable from clinically feasible EEG recordings without requiring behavioral responses from the patient, thereby circumventing the motor dependence and inter-rater variability of behavioral assessment. Second, the discriminative information is concentrated in a single parsimonious marker, which more complex multivariable models did not outperform, favoring an index that is easier to deploy and less prone to overfitting; because model development and evaluation were performed within the same modest-sized cohort, these figures represent internally rather than externally validated performance, and prospective validation in independent, multi-center cohorts is required before any diagnostic threshold can be proposed. Third, each marker is anchored to an anatomically defined network state—MS3 to right prefrontal control regions and MS4 to posterior integration hubs—offering circuit-level interpretability that complements recent large-cohort clinical-EEG screening models optimized for scalable screening rather than circuit-level targeting (Mou et al., 2026). The paradigm itself is readily translatable to neurointensive care and rehabilitation settings: auditory stimuli can be delivered through standard headphones with sound-insulating earmuffs, requiring no motor response, which expands the assessment repertoire for patients unable to perform motor imagery or command-following tasks (Bodien et al., 2024; Monti et al., 2010); at present, both stimulus types should be retained until condition equivalence is formally established. The full protocol (10-min rest plus approximately 30-min auditory stimulation) proved feasible at the bedside: in-ear earphones worn under circumaural sound-insulating earmuffs (3 M PELTOR X5A) were well tolerated, and no recording was terminated because of the setup. When vigilance fluctuated during a block, the operator logged the event and re-recorded that block after re-alerting the patient; residual low-vigilance segments were excluded during preprocessing. Whether an abbreviated protocol, such as the resting segment alone, retains comparable discriminative information remains to be tested directly; the absence of significant source-level condition effects is insufficient to establish this.

If confirmed, the candidate pathway could eventually inform circuit-level targets for precision neuromodulation—although we emphasize that this proposal is speculative, as it rests on a single connection that did not survive network-level correction and on Granger causality, whose directionality does not equate to physiological causality. Current non-invasive brain stimulation in DoC typically employs broad anatomical targets without circuit guidance (Wan et al., 2024), and efficacy appears to depend on preserved network architecture (Boly et al., 2017). Should this axis be validated—ideally through perturbational approaches such as TMS-EEG—strategies enhancing temporal-to-precuneus transmission might outperform generic whole-network approaches in patients with structurally preserved but functionally suppressed pathways. Finally, these biomarkers are not intended to replace CRS-R but to complement it. Behavioral scales primarily index arousal and motor responsiveness—dimensions closer to prefrontal function (Boly et al., 2017)—whereas the temporal-to-precuneus connectivity metric directly assesses the integrity of posterior sensory integration, a core component of the awareness dimension (Bayne et al., 2016). Given that cognitive motor dissociation is more prevalent than previously recognized (Bodien et al., 2024), behavioral assessment alone may overestimate consciousness level when arousal markers dissociate from awareness markers (Wang et al., 2022), and integrating EEG connectivity markers with CRS-R could reduce this diagnostic gap.

The opponent MS3/MS4 pattern maps onto the arousal–awareness distinction (Bodien et al., 2024; Monti et al., 2010): MS3 is dominated by right prefrontal regions tied to arousal and motor responsiveness—the dimensions indexed by behavioral scales—whereas MS4 loads on posterior integration hubs more closely associated with awareness per se. This mapping yields a falsifiable prediction: MS4 parameters should track CRS-R auditory and language subscales more closely than MS3 parameters. It likewise suggests that the MS4 elevation and the temporal-to-precuneus signal may be driven disproportionately by MCS + patients, in whom language function is preserved (Gui et al., 2020); our sample was too small to resolve this. Finally, the MS3-to-MS4 balance is a candidate longitudinal marker: if recovery entails restoring posterior integration relative to prefrontal predominance, this balance should track—and perhaps anticipate—behavioral recovery, which longitudinal EEG and CRS-R studies can test.

Three features of the present analysis may inform future work in cohorts of comparable size: parsimony outperformed complexity, suggesting that in small cohorts low-dimensional robust markers should be preferred over high-dimensional fragile ones; source resolution was decisive, with sensor-level microstate metrics failing to distinguish VS/UWS from MCS in the same cohort in which source-resolved metrics succeeded—a direct within-cohort demonstration that scalp-level analyses can obscure consciousness-level information; and transparent multiplicity reporting, testing the full set of 30 directed connections with false discovery rate correction and reporting the network-level null alongside the single nominally significant edge, offers a template for avoiding selective reporting in exploratory connectivity analyses.

### 4.5 Limitations

This study has several limitations. The sample size was relatively limited (17 VS/UWS and 16 MCS patients), and performance was evaluated within a single modest-sized cohort with wide confidence intervals. Although prediction-stage preprocessing and tuning were confined to training folds, template construction and MS3/MS4 selection used the present cohort before cross-validation. Consequently, residual optimism remains possible and the entire biomarker-discovery pipeline has not been independently validated; external, prospective validation in independent, multi-center cohorts with diverse etiologies is essential. None of the 30 directed Granger causality connections survived false discovery rate correction, and Granger causality directionality does not equate to physiological causality; concurrent TMS-EEG perturbation studies would strengthen causal inference. A further methodological dependency should be noted: the six regions entering the Granger causality analysis were selected on the basis of their contribution to source microstates 3 and 4, so the connectivity analysis and the microstate analysis are not fully independent in their node definition. Three considerations mitigate, but do not eliminate, this concern. First, Granger causality was computed on the continuous source-reconstructed time series, not on microstate-segmented data; the microstate maps served only to anchor region selection anatomically. Second, the selected nodes are themselves predefined anatomical parcels of the AAL atlas rather than arbitrary data-derived regions. Third, estimating connectivity across the whole AAL parcellation would have multiplied the number of directed edges by two orders of magnitude and thereby severely aggravated the multiple-comparison burden; the prespecified-ROI approach was chosen precisely to control false positives in a small cohort. Future studies should nevertheless seek to replicate the candidate pathway using region definitions that are fully independent of the microstate analysis, for example ROIs derived from task-based localizers, structural connectivity, or independent cohorts. In addition, MCS was not subdivided into MCS + and MCS − subtypes, source localization used a standardized head model rather than individual anatomical MRI, and the cross-sectional design precludes establishing longitudinal predictive value. Finally, the absence of statistically detectable differences between noise and speech conditions does not establish condition equivalence and should be tested in larger samples. The inclusion of both traumatic and cerebrovascular etiologies and a time since injury ranging from 1 to 12 months introduces clinical heterogeneity; microstate markers may differ by etiology and disease stage, which the present sample is too small to resolve. Non-significant baseline comparisons do not exclude residual confounding, and no covariate-adjusted EEG analysis was performed. In addition, local Holm correction does not control multiplicity across all microstate classes and metrics, so individual class-by-metric findings require independent replication.

## 5 Conclusion

This study presents a clinically oriented neurophysiological framework for objective consciousness-level assessment in disorders of consciousness. By integrating source-level EEG microstate analysis with a multi-condition auditory paradigm and Granger causality, we identified two opposing neurodynamic phenotypes—prefrontal entrapment with posterior disintegration in VS/UWS versus prefrontal release with posterior integration in MCS—that discriminated consciousness levels with moderate internally cross-validated accuracy (MS4 occurrence AUC = 0.812). Exploratory connectivity analysis across 30 directed edges provided preliminary, hypothesis-generating evidence implicating the pathway from the right middle temporal gyrus to the right precuneus. Of the two prespecified hypotheses, the state-level hypothesis was supported by five of the six MS3/MS4 temporal-parameter contrasts, whereas the circuit-level hypothesis was not confirmed under multiplicity control and remains to be tested in larger cohorts.

External validation in prospective, multi-center cohorts is the necessary next step before these markers can complement behavioral assessment or guide precision neuromodulation targeting in severely brain-injured patients.

## Data Availability

The raw data supporting the conclusions of this article will be made available by the authors, without undue reservation.
