# Supplementary material for: Source-resolved electroencephalogram microstates reveal opposing prefrontal-posterior dynamics and a candidate temporal-to-precuneus pathway in disorders of consciousness
Source: Front Neurosci. 2026 Oct 28;20:1995232. doi: 10.3389/fnins.2026.1995232 (PMC13751360; doi:10.3389/fnins.2026.1995232)
Supplement: Supplementary file 1 [file Supplementary_file_1.zip › Table 1.DOCX]

**Supplementary Statistical Tables and Figure**

Figure S1. Sensor-level microstate metrics across auditory conditions (rest, white noise, speech) for microstates A–E. (A) Mean duration in milliseconds, (B) coverage as a percentage, (C) occurrence rate per second. Raincloud plots combine half-violin density, box plots (interquartile range and median), and individual data points. Brackets denote Holm-adjusted post hoc pairwise comparisons: *p < .05, **p < .01, ***p < .001. N = 52 (HC = 19, MCS = 16, VS/UWS = 17). Pooled condition contrasts for class E coverage are exploratory (GG-corrected Condition p = .057); see Tables S7–S9 for interaction and simple-effect analyses.

**Sensor-level Results**

These supplementary tables report Holm-adjusted post hoc contrasts and Condition main effects from the prespecified Type III mixed repeated-measures analyses. For completeness and transparency, all pairwise comparisons are presented in these supplementary tables. Confirmatory interpretation is restricted to Holm-adjusted comparisons following a significant corresponding omnibus main effect. Pairwise comparisons without a significant corresponding omnibus effect are reported for completeness only and should be regarded as exploratory rather than independent confirmatory evidence. Group × Condition interactions and their corresponding simple-effect analyses are reported separately. Throughout the supplementary tables, duration is expressed in milliseconds, coverage as a percentage, and occurrence rate per second. VS denotes VS/UWS; Rest, REST, and resting refer to the same condition.

**Table S1**

*Sensor: Three-Group Post Hoc Pairwise Comparisons*

| **Metric** | **Class** | **Comparison** | **Mean1** | **SE1** | **Mean2** | **SE2** | **t(df)** | **Value of p** |
| --- | --- | --- | --- | --- | --- | --- | --- | --- |
| Duration | A | HC vs MCS | 29.565 | 1.889 | 40.932 | 2.058 | t(49) = -4.070 | < .001*** |
| Duration | A | HC vs VS | 29.565 | 1.889 | 42.882 | 1.997 | t(49) = -4.846 | < .001*** |
| Duration | A | MCS vs VS | 40.932 | 2.058 | 42.882 | 1.997 | t(49) = -0.680 | .500 |
| Duration | B | HC vs MCS | 28.912 | 1.958 | 40.743 | 2.134 | t(49) = -4.085 | < .001*** |
| Duration | B | HC vs VS | 28.912 | 1.958 | 41.061 | 2.070 | t(49) = -4.263 | < .001*** |
| Duration | B | MCS vs VS | 40.743 | 2.134 | 41.061 | 2.070 | t(49) = -0.107 | .915 |
| Duration | C | HC vs MCS | 32.309 | 1.899 | 41.431 | 2.069 | t(49) = -3.248 | .004** |
| Duration | C | HC vs VS | 32.309 | 1.899 | 42.925 | 2.008 | t(49) = -3.842 | .001** |
| Duration | C | MCS vs VS | 41.431 | 2.069 | 42.925 | 2.008 | t(49) = -0.518 | .607 |
| Duration | D | HC vs MCS | 29.327 | 1.948 | 37.348 | 2.123 | t(49) = -2.783 | .015* |
| Duration | D | HC vs VS | 29.327 | 1.948 | 40.805 | 2.060 | t(49) = -4.048 | < .001*** |
| Duration | D | MCS vs VS | 37.348 | 2.123 | 40.805 | 2.060 | t(49) = -1.169 | .248 |
| Duration | E | HC vs MCS | 29.112 | 1.925 | 40.212 | 2.098 | t(49) = -3.899 | < .001*** |
| Duration | E | HC vs VS | 29.112 | 1.925 | 43.397 | 2.035 | t(49) = -5.100 | < .001*** |
| Duration | E | MCS vs VS | 40.212 | 2.098 | 43.397 | 2.035 | t(49) = -1.090 | .281 |
| Occurrence | A | HC vs MCS | 6.784 | 0.396 | 5.741 | 0.432 | t(49) = 1.779 | .185 |
| Occurrence | A | HC vs VS | 6.784 | 0.396 | 5.681 | 0.419 | t(49) = 1.912 | .185 |
| Occurrence | A | MCS vs VS | 5.741 | 0.432 | 5.681 | 0.419 | t(49) = 0.100 | .921 |
| Occurrence | B | HC vs MCS | 7.406 | 0.397 | 5.726 | 0.433 | t(49) = 2.860 | .016* |
| Occurrence | B | HC vs VS | 7.406 | 0.397 | 5.724 | 0.420 | t(49) = 2.910 | .016* |
| Occurrence | B | MCS vs VS | 5.726 | 0.433 | 5.724 | 0.420 | t(49) = 0.002 | .998 |
| Occurrence | C | HC vs MCS | 8.039 | 0.395 | 5.841 | 0.430 | t(49) = 3.767 | < .001*** |
| Occurrence | C | HC vs VS | 8.039 | 0.395 | 5.468 | 0.417 | t(49) = 4.478 | < .001*** |
| Occurrence | C | MCS vs VS | 5.841 | 0.430 | 5.468 | 0.417 | t(49) = 0.623 | .536 |
| Occurrence | D | HC vs MCS | 6.740 | 0.410 | 4.967 | 0.446 | t(49) = 2.927 | .010* |
| Occurrence | D | HC vs VS | 6.740 | 0.410 | 4.631 | 0.433 | t(49) = 3.539 | .003** |
| Occurrence | D | MCS vs VS | 4.967 | 0.446 | 4.631 | 0.433 | t(49) = 0.540 | .592 |
| Occurrence | E | HC vs MCS | 6.673 | 0.380 | 5.073 | 0.414 | t(49) = 2.845 | .013* |
| Occurrence | E | HC vs VS | 6.673 | 0.380 | 4.931 | 0.402 | t(49) = 3.149 | .008** |
| Occurrence | E | MCS vs VS | 5.073 | 0.414 | 4.931 | 0.402 | t(49) = 0.247 | .806 |
| Coverage | A | HC vs MCS | 18.360 | 0.819 | 21.635 | 0.892 | t(49) = -2.705 | .019* |
| Coverage | A | HC vs VS | 18.360 | 0.819 | 21.855 | 0.865 | t(49) = -2.934 | .015* |
| Coverage | A | MCS vs VS | 21.635 | 0.892 | 21.855 | 0.865 | t(49) = -0.177 | .860 |
| Coverage | B | HC vs MCS | 20.292 | 0.720 | 21.234 | 0.785 | t(49) = -0.884 | 1.000 |
| Coverage | B | HC vs VS | 20.292 | 0.720 | 20.943 | 0.762 | t(49) = -0.621 | 1.000 |
| Coverage | B | MCS vs VS | 21.234 | 0.785 | 20.943 | 0.762 | t(49) = 0.266 | 1.000 |
| Coverage | C | HC vs MCS | 24.684 | 0.969 | 21.777 | 1.055 | t(49) = 2.029 | .096 |
| Coverage | C | HC vs VS | 24.684 | 0.969 | 20.783 | 1.024 | t(49) = 2.768 | .024* |
| Coverage | C | MCS vs VS | 21.777 | 1.055 | 20.783 | 1.024 | t(49) = 0.676 | .502 |
| Coverage | D | HC vs MCS | 18.458 | 0.934 | 16.720 | 1.017 | t(49) = 1.259 | .642 |
| Coverage | D | HC vs VS | 18.458 | 0.934 | 16.952 | 0.987 | t(49) = 1.109 | .642 |
| Coverage | D | MCS vs VS | 16.720 | 1.017 | 16.952 | 0.987 | t(49) = -0.164 | .871 |
| Coverage | E | HC vs MCS | 18.206 | 0.926 | 18.634 | 1.009 | t(49) = -0.313 | 1.000 |
| Coverage | E | HC vs VS | 18.206 | 0.926 | 19.467 | 0.978 | t(49) = -0.937 | 1.000 |
| Coverage | E | MCS vs VS | 18.634 | 1.009 | 19.467 | 0.978 | t(49) = -0.593 | 1.000 |

Note. SE = standard error. Pairwise contrasts are Holm-adjusted post hoc comparisons from the full three-group mixed model. *p < .05. **p < .01. ***p < .001.

**Table S2**

*Sensor: Condition Effect*

| **Metric** | **Class** | **Rest Mean** | **Rest SE** | **Noise Mean** | **Noise SE** | **Speech Mean** | **Speech SE** | **F(df1, df2)** | **Value of p** | **partial η²** |
| --- | --- | --- | --- | --- | --- | --- | --- | --- | --- | --- |
| Duration | A | 31.820 | 1.511 | 40.889 | 1.701 | 40.671 | 1.502 | F(1.82, 89.28) = 15.288 | < .001*** | 0.238 |
| Duration | B | 31.748 | 1.614 | 38.884 | 1.430 | 40.084 | 1.575 | F(1.57, 77.13) = 13.982 | < .001*** | 0.222 |
| Duration | C | 31.925 | 1.702 | 41.590 | 1.452 | 43.149 | 1.696 | F(1.66, 81.52) = 18.913 | < .001*** | 0.278 |
| Duration | D | 28.758 | 1.518 | 38.460 | 1.662 | 40.261 | 1.538 | F(1.69, 82.76) = 23.558 | < .001*** | 0.325 |
| Duration | E | 31.186 | 1.651 | 41.049 | 1.540 | 40.486 | 1.629 | F(1.69, 82.85) = 16.726 | < .001*** | 0.254 |
| Occurrence | A | 7.487 | 0.466 | 5.224 | 0.309 | 5.493 | 0.263 | F(1.35, 66.34) = 14.665 | < .001*** | 0.230 |
| Occurrence | B | 7.929 | 0.480 | 5.511 | 0.282 | 5.416 | 0.300 | F(1.41, 69.06) = 17.917 | < .001*** | 0.268 |
| Occurrence | C | 8.035 | 0.480 | 5.842 | 0.251 | 5.470 | 0.296 | F(1.35, 66.33) = 18.295 | < .001*** | 0.272 |
| Occurrence | D | 6.782 | 0.537 | 4.591 | 0.292 | 4.964 | 0.344 | F(1.64, 80.35) = 8.955 | < .001*** | 0.155 |
| Occurrence | E | 6.985 | 0.481 | 5.275 | 0.219 | 4.417 | 0.331 | F(1.61, 78.70) = 14.840 | < .001*** | 0.232 |
| Coverage | A | 20.604 | 0.694 | 20.149 | 0.900 | 21.096 | 0.782 | F(1.94, 95.22) = 0.385 | .675 | 0.008 |
| Coverage | B | 21.945 | 0.765 | 19.931 | 0.756 | 20.593 | 0.939 | F(1.74, 85.04) = 1.438 | .243 | 0.029 |
| Coverage | C | 21.968 | 0.610 | 23.081 | 0.907 | 22.196 | 1.162 | F(1.88, 91.94) = 0.457 | .622 | 0.009 |
| Coverage | D | 16.596 | 0.834 | 16.607 | 0.998 | 18.928 | 1.086 | F(1.92, 94.20) = 1.888 | .159 | 0.037 |
| Coverage | E | 18.887 | 0.816 | 20.233 | 0.741 | 17.187 | 1.102 | F(1.69, 82.74) = 3.133 | .057 | 0.060 |

Note. SE = standard error; partial η² = partial eta squared. The table reports the Condition main effect across Rest, Noise, and Speech from the prespecified Type III mixed repeated-measures model. F tests use Greenhouse–Geisser-corrected degrees of freedom and p values. *p < .05. **p < .01. ***p < .001.

**Table S3**

*Sensor: Condition Post Hoc Pairwise Comparisons*

| **Metric** | **Class** | **Comparison** | **Mean1** | **SE1** | **Mean2** | **SE2** | **t(df)** | **Value of p** |
| --- | --- | --- | --- | --- | --- | --- | --- | --- |
| Duration | A | Rest vs Noise | 31.820 | 1.511 | 40.889 | 1.701 | t(49) = -4.395 | < .001*** |
| Duration | A | Rest vs Speech | 31.820 | 1.511 | 40.671 | 1.502 | t(49) = -4.533 | < .001*** |
| Duration | A | Noise vs Speech | 40.889 | 1.701 | 40.671 | 1.502 | t(49) = 0.139 | .890 |
| Duration | B | Rest vs Noise | 31.748 | 1.614 | 38.884 | 1.430 | t(49) = -4.365 | < .001*** |
| Duration | B | Rest vs Speech | 31.748 | 1.614 | 40.084 | 1.575 | t(49) = -4.013 | < .001*** |
| Duration | B | Noise vs Speech | 38.884 | 1.430 | 40.084 | 1.575 | t(49) = -0.913 | .366 |
| Duration | C | Rest vs Noise | 31.925 | 1.702 | 41.590 | 1.452 | t(49) = -4.550 | < .001*** |
| Duration | C | Rest vs Speech | 31.925 | 1.702 | 43.149 | 1.696 | t(49) = -5.000 | < .001*** |
| Duration | C | Noise vs Speech | 41.590 | 1.452 | 43.149 | 1.696 | t(49) = -1.056 | .296 |
| Duration | D | Rest vs Noise | 28.758 | 1.518 | 38.460 | 1.662 | t(49) = -5.256 | < .001*** |
| Duration | D | Rest vs Speech | 28.758 | 1.518 | 40.261 | 1.538 | t(49) = -5.502 | < .001*** |
| Duration | D | Noise vs Speech | 38.460 | 1.662 | 40.261 | 1.538 | t(49) = -1.283 | .205 |
| Duration | E | Rest vs Noise | 31.186 | 1.651 | 41.049 | 1.540 | t(49) = -4.705 | < .001*** |
| Duration | E | Rest vs Speech | 31.186 | 1.651 | 40.486 | 1.629 | t(49) = -4.379 | < .001*** |
| Duration | E | Noise vs Speech | 41.049 | 1.540 | 40.486 | 1.629 | t(49) = 0.389 | .699 |
| Occurrence | A | Rest vs Noise | 7.487 | 0.466 | 5.224 | 0.309 | t(49) = 4.295 | < .001*** |
| Occurrence | A | Rest vs Speech | 7.487 | 0.466 | 5.493 | 0.263 | t(49) = 3.747 | < .001*** |
| Occurrence | A | Noise vs Speech | 5.224 | 0.309 | 5.493 | 0.263 | t(49) = -1.058 | .295 |
| Occurrence | B | Rest vs Noise | 7.929 | 0.480 | 5.511 | 0.282 | t(49) = 4.258 | < .001*** |
| Occurrence | B | Rest vs Speech | 7.929 | 0.480 | 5.416 | 0.300 | t(49) = 4.798 | < .001*** |
| Occurrence | B | Noise vs Speech | 5.511 | 0.282 | 5.416 | 0.300 | t(49) = 0.328 | .744 |
| Occurrence | C | Rest vs Noise | 8.035 | 0.480 | 5.842 | 0.251 | t(49) = 4.216 | < .001*** |
| Occurrence | C | Rest vs Speech | 8.035 | 0.480 | 5.470 | 0.296 | t(49) = 4.730 | < .001*** |
| Occurrence | C | Noise vs Speech | 5.842 | 0.251 | 5.470 | 0.296 | t(49) = 1.455 | .152 |
| Occurrence | D | Rest vs Noise | 6.782 | 0.537 | 4.591 | 0.292 | t(49) = 3.465 | .003** |
| Occurrence | D | Rest vs Speech | 6.782 | 0.537 | 4.964 | 0.344 | t(49) = 3.050 | .007** |
| Occurrence | D | Noise vs Speech | 4.591 | 0.292 | 4.964 | 0.344 | t(49) = -0.915 | .365 |
| Occurrence | E | Rest vs Noise | 6.985 | 0.481 | 5.275 | 0.219 | t(49) = 3.310 | .004** |
| Occurrence | E | Rest vs Speech | 6.985 | 0.481 | 4.417 | 0.331 | t(49) = 4.645 | < .001*** |
| Occurrence | E | Noise vs Speech | 5.275 | 0.219 | 4.417 | 0.331 | t(49) = 2.491 | .016* |
| Coverage | A | Rest vs Noise | 20.604 | 0.694 | 20.149 | 0.900 | t(49) = 0.406 | 1.000 |
| Coverage | A | Rest vs Speech | 20.604 | 0.694 | 21.096 | 0.782 | t(49) = -0.437 | 1.000 |
| Coverage | A | Noise vs Speech | 20.149 | 0.900 | 21.096 | 0.782 | t(49) = -0.964 | 1.000 |
| Coverage | B | Rest vs Noise | 21.945 | 0.765 | 19.931 | 0.756 | t(49) = 1.605 | .345 |
| Coverage | B | Rest vs Speech | 21.945 | 0.765 | 20.593 | 0.939 | t(49) = 0.982 | .662 |
| Coverage | B | Noise vs Speech | 19.931 | 0.756 | 20.593 | 0.939 | t(49) = -0.688 | .662 |
| Coverage | C | Rest vs Noise | 21.968 | 0.610 | 23.081 | 0.907 | t(49) = -1.044 | .905 |
| Coverage | C | Rest vs Speech | 21.968 | 0.610 | 22.196 | 1.162 | t(49) = -0.179 | 1.000 |
| Coverage | C | Noise vs Speech | 23.081 | 0.907 | 22.196 | 1.162 | t(49) = 0.662 | 1.000 |
| Coverage | D | Rest vs Noise | 16.596 | 0.834 | 16.607 | 0.998 | t(49) = -0.008 | .993 |
| Coverage | D | Rest vs Speech | 16.596 | 0.834 | 18.928 | 1.086 | t(49) = -1.714 | .279 |
| Coverage | D | Noise vs Speech | 16.607 | 0.998 | 18.928 | 1.086 | t(49) = -1.541 | .279 |
| Coverage | E | Rest vs Noise | 18.887 | 0.816 | 20.233 | 0.741 | t(49) = -1.312 | .391 |
| Coverage | E | Rest vs Speech | 18.887 | 0.816 | 17.187 | 1.102 | t(49) = 1.170 | .391 |
| Coverage | E | Noise vs Speech | 20.233 | 0.741 | 17.187 | 1.102 | t(49) = 2.673 | .031* |

Note. SE = standard error. Pairwise contrasts are Holm-adjusted estimated marginal mean comparisons for Condition from the prespecified Type III mixed repeated-measures model. The t ratio is reported for the first condition minus the second condition. *p < .05. **p < .01. ***p < .001.

**Source-level Results**

**Table S4**

*Source: Three-Group Post Hoc Pairwise Comparisons*

| **Metric** | **Class** | **Comparison** | **Mean1** | **SE1** | **Mean2** | **SE2** | **t(df)** | **Value of p** |
| --- | --- | --- | --- | --- | --- | --- | --- | --- |
| Duration | 1 | HC vs MCS | 24.895 | 2.469 | 34.552 | 2.691 | t(49) = -2.644 | .022* |
| Duration | 1 | HC vs VS | 24.895 | 2.469 | 34.921 | 2.611 | t(49) = -2.790 | .022* |
| Duration | 1 | MCS vs VS | 34.552 | 2.691 | 34.921 | 2.611 | t(49) = -0.098 | .922 |
| Duration | 2 | HC vs MCS | 21.663 | 2.557 | 34.278 | 2.787 | t(49) = -3.335 | .003** |
| Duration | 2 | HC vs VS | 21.663 | 2.557 | 35.381 | 2.703 | t(49) = -3.687 | .002** |
| Duration | 2 | MCS vs VS | 34.278 | 2.787 | 35.381 | 2.703 | t(49) = -0.284 | .777 |
| Duration | 3 | HC vs MCS | 21.286 | 2.991 | 32.354 | 3.259 | t(49) = -2.502 | .031* |
| Duration | 3 | HC vs VS | 21.286 | 2.991 | 42.134 | 3.162 | t(49) = -4.790 | < .001*** |
| Duration | 3 | MCS vs VS | 32.354 | 3.259 | 42.134 | 3.162 | t(49) = -2.154 | .036* |
| Duration | 4 | HC vs MCS | 22.394 | 2.880 | 40.291 | 3.139 | t(49) = -4.201 | < .001*** |
| Duration | 4 | HC vs VS | 22.394 | 2.880 | 32.697 | 3.045 | t(49) = -2.458 | .035* |
| Duration | 4 | MCS vs VS | 40.291 | 3.139 | 32.697 | 3.045 | t(49) = 1.737 | .089 |
| Duration | 5 | HC vs MCS | 22.362 | 1.783 | 30.300 | 1.943 | t(49) = -3.009 | .008** |
| Duration | 5 | HC vs VS | 22.362 | 1.783 | 32.894 | 1.885 | t(49) = -4.058 | < .001*** |
| Duration | 5 | MCS vs VS | 30.300 | 1.943 | 32.894 | 1.885 | t(49) = -0.958 | .343 |
| Duration | 6 | HC vs MCS | 25.528 | 2.028 | 36.271 | 2.210 | t(49) = -3.582 | .002** |
| Duration | 6 | HC vs VS | 25.528 | 2.028 | 31.221 | 2.144 | t(49) = -1.929 | .119 |
| Duration | 6 | MCS vs VS | 36.271 | 2.210 | 31.221 | 2.144 | t(49) = 1.640 | .119 |
| Duration | 7 | HC vs MCS | 21.163 | 2.061 | 31.589 | 2.246 | t(49) = -3.420 | .003** |
| Duration | 7 | HC vs VS | 21.163 | 2.061 | 35.525 | 2.179 | t(49) = -4.788 | < .001*** |
| Duration | 7 | MCS vs VS | 31.589 | 2.246 | 35.525 | 2.179 | t(49) = -1.257 | .215 |
| Occurrence | 1 | HC vs MCS | 7.617 | 0.555 | 4.802 | 0.604 | t(49) = 3.432 | .002** |
| Occurrence | 1 | HC vs VS | 7.617 | 0.555 | 4.261 | 0.586 | t(49) = 4.159 | < .001*** |
| Occurrence | 1 | MCS vs VS | 4.802 | 0.604 | 4.261 | 0.586 | t(49) = 0.643 | .523 |
| Occurrence | 2 | HC vs MCS | 4.333 | 0.489 | 2.026 | 0.533 | t(49) = 3.190 | .007** |
| Occurrence | 2 | HC vs VS | 4.333 | 0.489 | 2.618 | 0.517 | t(49) = 2.410 | .039* |
| Occurrence | 2 | MCS vs VS | 2.026 | 0.533 | 2.618 | 0.517 | t(49) = -0.797 | .429 |
| Occurrence | 3 | HC vs MCS | 5.071 | 0.559 | 2.835 | 0.609 | t(49) = 2.705 | .019* |
| Occurrence | 3 | HC vs VS | 5.071 | 0.559 | 5.430 | 0.591 | t(49) = -0.442 | .661 |
| Occurrence | 3 | MCS vs VS | 2.835 | 0.609 | 5.430 | 0.591 | t(49) = -3.058 | .011* |
| Occurrence | 4 | HC vs MCS | 3.658 | 0.467 | 3.028 | 0.508 | t(49) = 0.914 | .365 |
| Occurrence | 4 | HC vs VS | 3.658 | 0.467 | 0.845 | 0.493 | t(49) = 4.143 | < .001*** |
| Occurrence | 4 | MCS vs VS | 3.028 | 0.508 | 0.845 | 0.493 | t(49) = 3.081 | .007** |
| Occurrence | 5 | HC vs MCS | 6.742 | 0.545 | 3.465 | 0.593 | t(49) = 4.067 | < .001*** |
| Occurrence | 5 | HC vs VS | 6.742 | 0.545 | 4.366 | 0.576 | t(49) = 2.997 | .009** |
| Occurrence | 5 | MCS vs VS | 3.465 | 0.593 | 4.366 | 0.576 | t(49) = -1.090 | .281 |
| Occurrence | 6 | HC vs MCS | 8.737 | 0.656 | 5.625 | 0.714 | t(49) = 3.210 | .005** |
| Occurrence | 6 | HC vs VS | 8.737 | 0.656 | 4.522 | 0.693 | t(49) = 4.418 | < .001*** |
| Occurrence | 6 | MCS vs VS | 5.625 | 0.714 | 4.522 | 0.693 | t(49) = 1.108 | .273 |
| Occurrence | 7 | HC vs MCS | 5.474 | 0.675 | 3.726 | 0.736 | t(49) = 1.751 | .259 |
| Occurrence | 7 | HC vs VS | 5.474 | 0.675 | 4.638 | 0.714 | t(49) = 0.851 | .756 |
| Occurrence | 7 | MCS vs VS | 3.726 | 0.736 | 4.638 | 0.714 | t(49) = -0.890 | .756 |
| Coverage | 1 | HC vs MCS | 19.721 | 2.334 | 18.119 | 2.543 | t(49) = 0.464 | .836 |
| Coverage | 1 | HC vs VS | 19.721 | 2.334 | 15.224 | 2.467 | t(49) = 1.324 | .575 |
| Coverage | 1 | MCS vs VS | 18.119 | 2.543 | 15.224 | 2.467 | t(49) = 0.817 | .836 |
| Coverage | 2 | HC vs MCS | 9.836 | 2.273 | 8.441 | 2.477 | t(49) = 0.415 | 1.000 |
| Coverage | 2 | HC vs VS | 9.836 | 2.273 | 9.205 | 2.403 | t(49) = 0.191 | 1.000 |
| Coverage | 2 | MCS vs VS | 8.441 | 2.477 | 9.205 | 2.403 | t(49) = -0.222 | 1.000 |
| Coverage | 3 | HC vs MCS | 10.916 | 2.965 | 11.096 | 3.231 | t(49) = -0.041 | .967 |
| Coverage | 3 | HC vs VS | 10.916 | 2.965 | 25.172 | 3.135 | t(49) = -3.304 | .005** |
| Coverage | 3 | MCS vs VS | 11.096 | 3.231 | 25.172 | 3.135 | t(49) = -3.126 | .006** |
| Coverage | 4 | HC vs MCS | 8.801 | 2.649 | 15.338 | 2.887 | t(49) = -1.668 | .203 |
| Coverage | 4 | HC vs VS | 8.801 | 2.649 | 3.356 | 2.801 | t(49) = 1.412 | .203 |
| Coverage | 4 | MCS vs VS | 15.338 | 2.887 | 3.356 | 2.801 | t(49) = 2.979 | .013* |
| Coverage | 5 | HC vs MCS | 15.591 | 1.807 | 11.467 | 1.970 | t(49) = 1.543 | .388 |
| Coverage | 5 | HC vs VS | 15.591 | 1.807 | 14.893 | 1.911 | t(49) = 0.265 | .792 |
| Coverage | 5 | MCS vs VS | 11.467 | 1.970 | 14.893 | 1.911 | t(49) = -1.249 | .436 |
| Coverage | 6 | HC vs MCS | 23.425 | 3.037 | 22.677 | 3.310 | t(49) = 0.167 | .868 |
| Coverage | 6 | HC vs VS | 23.425 | 3.037 | 14.966 | 3.211 | t(49) = 1.914 | .184 |
| Coverage | 6 | MCS vs VS | 22.677 | 3.310 | 14.966 | 3.211 | t(49) = 1.672 | .202 |
| Coverage | 7 | HC vs MCS | 11.711 | 2.715 | 12.862 | 2.958 | t(49) = -0.287 | .775 |
| Coverage | 7 | HC vs VS | 11.711 | 2.715 | 17.183 | 2.870 | t(49) = -1.385 | .517 |
| Coverage | 7 | MCS vs VS | 12.862 | 2.958 | 17.183 | 2.870 | t(49) = -1.048 | .599 |

Note. SE = standard error. Pairwise contrasts are Holm-adjusted post hoc comparisons from the full three-group mixed model. *p < .05. **p < .01. ***p < .001.

**Table S5**

*Source: Condition Effect*

| **Metric** | **Class** | **Rest Mean** | **Rest SE** | **Noise Mean** | **Noise SE** | **Speech Mean** | **Speech SE** | **F(df1, df2)** | **Value of p** | **partial η²** |
| --- | --- | --- | --- | --- | --- | --- | --- | --- | --- | --- |
| Duration | 1 | 32.790 | 3.015 | 29.492 | 1.279 | 32.087 | 1.870 | F(1.30, 63.54) = 0.805 | .403 | 0.016 |
| Duration | 2 | 27.831 | 1.077 | 31.034 | 2.736 | 32.457 | 2.248 | F(1.63, 79.81) = 1.728 | .189 | 0.034 |
| Duration | 3 | 31.524 | 1.896 | 31.281 | 1.957 | 32.969 | 2.171 | F(1.74, 85.23) = 0.730 | .467 | 0.015 |
| Duration | 4 | 29.784 | 1.260 | 33.586 | 2.934 | 32.012 | 2.303 | F(1.68, 82.55) = 1.148 | .315 | 0.023 |
| Duration | 5 | 29.037 | 1.369 | 27.814 | 1.321 | 28.705 | 1.171 | F(1.19, 58.20) = 0.538 | .496 | 0.011 |
| Duration | 6 | 30.550 | 1.320 | 30.988 | 1.480 | 31.482 | 1.478 | F(1.68, 82.43) = 0.273 | .723 | 0.006 |
| Duration | 7 | 29.733 | 1.398 | 29.318 | 1.654 | 29.226 | 1.389 | F(1.22, 59.88) = 0.075 | .833 | 0.002 |
| Occurrence | 1 | 5.722 | 0.400 | 5.566 | 0.418 | 5.392 | 0.442 | F(1.61, 78.93) = 0.285 | .705 | 0.006 |
| Occurrence | 2 | 2.979 | 0.373 | 2.890 | 0.347 | 3.109 | 0.339 | F(1.36, 66.40) = 0.218 | .716 | 0.004 |
| Occurrence | 3 | 4.939 | 0.451 | 4.245 | 0.415 | 4.153 | 0.360 | F(1.38, 67.54) = 2.290 | .126 | 0.045 |
| Occurrence | 4 | 2.175 | 0.362 | 2.639 | 0.368 | 2.717 | 0.337 | F(1.69, 82.76) = 1.226 | .294 | 0.024 |
| Occurrence | 5 | 5.072 | 0.419 | 4.647 | 0.388 | 4.855 | 0.450 | F(1.63, 79.77) = 0.448 | .600 | 0.009 |
| Occurrence | 6 | 6.338 | 0.395 | 6.472 | 0.500 | 6.074 | 0.432 | F(1.55, 75.73) = 0.682 | .472 | 0.014 |
| Occurrence | 7 | 4.831 | 0.500 | 4.500 | 0.450 | 4.506 | 0.511 | F(1.79, 87.69) = 0.339 | .690 | 0.007 |
| Coverage | 1 | 18.633 | 1.913 | 16.574 | 1.642 | 17.858 | 2.120 | F(1.51, 73.91) = 0.446 | .586 | 0.009 |
| Coverage | 2 | 8.216 | 1.376 | 9.225 | 1.799 | 10.041 | 1.858 | F(1.25, 61.35) = 0.578 | .487 | 0.012 |
| Coverage | 3 | 16.668 | 2.097 | 15.078 | 2.195 | 15.438 | 1.958 | F(1.22, 59.60) = 0.414 | .562 | 0.008 |
| Coverage | 4 | 7.031 | 1.651 | 10.919 | 2.515 | 9.545 | 1.874 | F(1.43, 69.99) = 1.610 | .211 | 0.032 |
| Coverage | 5 | 14.946 | 1.661 | 13.353 | 1.337 | 13.652 | 1.272 | F(1.51, 73.95) = 0.558 | .527 | 0.011 |
| Coverage | 6 | 19.797 | 1.853 | 21.259 | 2.185 | 20.012 | 2.029 | F(1.64, 80.35) = 0.576 | .531 | 0.012 |
| Coverage | 7 | 14.709 | 1.958 | 13.593 | 1.866 | 13.455 | 1.815 | F(1.48, 72.43) = 0.380 | .622 | 0.008 |

Note. SE = standard error; partial η² = partial eta squared. The table reports the Condition main effect across Rest, Noise, and Speech from the prespecified Type III mixed repeated-measures model. F tests use Greenhouse–Geisser-corrected degrees of freedom and p values. *p < .05. **p < .01. ***p < .001.

**Table S6**

*Source: Condition Post Hoc Pairwise Comparisons*

| **Metric** | **Class** | **Comparison** | **Mean1** | **SE1** | **Mean2** | **SE2** | **t(df)** | **Value of p** |
| --- | --- | --- | --- | --- | --- | --- | --- | --- |
| Duration | 1 | Rest vs Noise | 32.790 | 3.015 | 29.492 | 1.279 | t(49) = 1.058 | .590 |
| Duration | 1 | Rest vs Speech | 32.790 | 3.015 | 32.087 | 1.870 | t(49) = 0.214 | .831 |
| Duration | 1 | Noise vs Speech | 29.492 | 1.279 | 32.087 | 1.870 | t(49) = -1.830 | .220 |
| Duration | 2 | Rest vs Noise | 27.831 | 1.077 | 31.034 | 2.736 | t(49) = -1.054 | .594 |
| Duration | 2 | Rest vs Speech | 27.831 | 1.077 | 32.457 | 2.248 | t(49) = -1.831 | .220 |
| Duration | 2 | Noise vs Speech | 31.034 | 2.736 | 32.457 | 2.248 | t(49) = -0.724 | .594 |
| Duration | 3 | Rest vs Noise | 31.524 | 1.896 | 31.281 | 1.957 | t(49) = 0.141 | .888 |
| Duration | 3 | Rest vs Speech | 31.524 | 1.896 | 32.969 | 2.171 | t(49) = -0.926 | .718 |
| Duration | 3 | Noise vs Speech | 31.281 | 1.957 | 32.969 | 2.171 | t(49) = -1.402 | .502 |
| Duration | 4 | Rest vs Noise | 29.784 | 1.260 | 33.586 | 2.934 | t(49) = -1.268 | .632 |
| Duration | 4 | Rest vs Speech | 29.784 | 1.260 | 32.012 | 2.303 | t(49) = -0.930 | .714 |
| Duration | 4 | Noise vs Speech | 33.586 | 2.934 | 32.012 | 2.303 | t(49) = 0.755 | .714 |
| Duration | 5 | Rest vs Noise | 29.037 | 1.369 | 27.814 | 1.321 | t(49) = 0.746 | .918 |
| Duration | 5 | Rest vs Speech | 29.037 | 1.369 | 28.705 | 1.171 | t(49) = 0.316 | .918 |
| Duration | 5 | Noise vs Speech | 27.814 | 1.321 | 28.705 | 1.171 | t(49) = -1.095 | .837 |
| Duration | 6 | Rest vs Noise | 30.550 | 1.320 | 30.988 | 1.480 | t(49) = -0.304 | 1.000 |
| Duration | 6 | Rest vs Speech | 30.550 | 1.320 | 31.482 | 1.478 | t(49) = -0.700 | 1.000 |
| Duration | 6 | Noise vs Speech | 30.988 | 1.480 | 31.482 | 1.478 | t(49) = -0.514 | 1.000 |
| Duration | 7 | Rest vs Noise | 29.733 | 1.398 | 29.318 | 1.654 | t(49) = 0.235 | 1.000 |
| Duration | 7 | Rest vs Speech | 29.733 | 1.398 | 29.226 | 1.389 | t(49) = 0.342 | 1.000 |
| Duration | 7 | Noise vs Speech | 29.318 | 1.654 | 29.226 | 1.389 | t(49) = 0.133 | 1.000 |
| Occurrence | 1 | Rest vs Noise | 5.722 | 0.400 | 5.566 | 0.418 | t(49) = 0.320 | 1.000 |
| Occurrence | 1 | Rest vs Speech | 5.722 | 0.400 | 5.392 | 0.442 | t(49) = 0.674 | 1.000 |
| Occurrence | 1 | Noise vs Speech | 5.566 | 0.418 | 5.392 | 0.442 | t(49) = 0.558 | 1.000 |
| Occurrence | 2 | Rest vs Noise | 2.979 | 0.373 | 2.890 | 0.347 | t(49) = 0.218 | 1.000 |
| Occurrence | 2 | Rest vs Speech | 2.979 | 0.373 | 3.109 | 0.339 | t(49) = -0.359 | 1.000 |
| Occurrence | 2 | Noise vs Speech | 2.890 | 0.347 | 3.109 | 0.339 | t(49) = -1.134 | .787 |
| Occurrence | 3 | Rest vs Noise | 4.939 | 0.451 | 4.245 | 0.415 | t(49) = 1.377 | .349 |
| Occurrence | 3 | Rest vs Speech | 4.939 | 0.451 | 4.153 | 0.360 | t(49) = 1.942 | .174 |
| Occurrence | 3 | Noise vs Speech | 4.245 | 0.415 | 4.153 | 0.360 | t(49) = 0.358 | .722 |
| Occurrence | 4 | Rest vs Noise | 2.175 | 0.362 | 2.639 | 0.368 | t(49) = -1.050 | .598 |
| Occurrence | 4 | Rest vs Speech | 2.175 | 0.362 | 2.717 | 0.337 | t(49) = -1.490 | .428 |
| Occurrence | 4 | Noise vs Speech | 2.639 | 0.368 | 2.717 | 0.337 | t(49) = -0.256 | .799 |
| Occurrence | 5 | Rest vs Noise | 5.072 | 0.419 | 4.647 | 0.388 | t(49) = 0.927 | 1.000 |
| Occurrence | 5 | Rest vs Speech | 5.072 | 0.419 | 4.855 | 0.450 | t(49) = 0.410 | 1.000 |
| Occurrence | 5 | Noise vs Speech | 4.647 | 0.388 | 4.855 | 0.450 | t(49) = -0.614 | 1.000 |
| Occurrence | 6 | Rest vs Noise | 6.338 | 0.395 | 6.472 | 0.500 | t(49) = -0.314 | .825 |
| Occurrence | 6 | Rest vs Speech | 6.338 | 0.395 | 6.074 | 0.432 | t(49) = 0.826 | .825 |
| Occurrence | 6 | Noise vs Speech | 6.472 | 0.500 | 6.074 | 0.432 | t(49) = 1.452 | .459 |
| Occurrence | 7 | Rest vs Noise | 4.831 | 0.500 | 4.500 | 0.450 | t(49) = 0.639 | 1.000 |
| Occurrence | 7 | Rest vs Speech | 4.831 | 0.500 | 4.506 | 0.511 | t(49) = 0.689 | 1.000 |
| Occurrence | 7 | Noise vs Speech | 4.500 | 0.450 | 4.506 | 0.511 | t(49) = -0.016 | 1.000 |
| Coverage | 1 | Rest vs Noise | 18.633 | 1.913 | 16.574 | 1.642 | t(49) = 0.902 | 1.000 |
| Coverage | 1 | Rest vs Speech | 18.633 | 1.913 | 17.858 | 2.120 | t(49) = 0.292 | 1.000 |
| Coverage | 1 | Noise vs Speech | 16.574 | 1.642 | 17.858 | 2.120 | t(49) = -0.849 | 1.000 |
| Coverage | 2 | Rest vs Noise | 8.216 | 1.376 | 9.225 | 1.799 | t(49) = -0.496 | .963 |
| Coverage | 2 | Rest vs Speech | 8.216 | 1.376 | 10.041 | 1.858 | t(49) = -0.927 | .963 |
| Coverage | 2 | Noise vs Speech | 9.225 | 1.799 | 10.041 | 1.858 | t(49) = -1.003 | .963 |
| Coverage | 3 | Rest vs Noise | 16.668 | 2.097 | 15.078 | 2.195 | t(49) = 0.697 | 1.000 |
| Coverage | 3 | Rest vs Speech | 16.668 | 2.097 | 15.438 | 1.958 | t(49) = 0.606 | 1.000 |
| Coverage | 3 | Noise vs Speech | 15.078 | 2.195 | 15.438 | 1.958 | t(49) = -0.420 | 1.000 |
| Coverage | 4 | Rest vs Noise | 7.031 | 1.651 | 10.919 | 2.515 | t(49) = -1.385 | .517 |
| Coverage | 4 | Rest vs Speech | 7.031 | 1.651 | 9.545 | 1.874 | t(49) = -1.349 | .517 |
| Coverage | 4 | Noise vs Speech | 10.919 | 2.515 | 9.545 | 1.874 | t(49) = 0.775 | .517 |
| Coverage | 5 | Rest vs Noise | 14.946 | 1.661 | 13.353 | 1.337 | t(49) = 0.865 | 1.000 |
| Coverage | 5 | Rest vs Speech | 14.946 | 1.661 | 13.652 | 1.272 | t(49) = 0.722 | 1.000 |
| Coverage | 5 | Noise vs Speech | 13.353 | 1.337 | 13.652 | 1.272 | t(49) = -0.284 | 1.000 |
| Coverage | 6 | Rest vs Noise | 19.797 | 1.853 | 21.259 | 2.185 | t(49) = -0.836 | .839 |
| Coverage | 6 | Rest vs Speech | 19.797 | 1.853 | 20.012 | 2.029 | t(49) = -0.148 | .883 |
| Coverage | 6 | Noise vs Speech | 21.259 | 2.185 | 20.012 | 2.029 | t(49) = 1.093 | .839 |
| Coverage | 7 | Rest vs Noise | 14.709 | 1.958 | 13.593 | 1.866 | t(49) = 0.604 | 1.000 |
| Coverage | 7 | Rest vs Speech | 14.709 | 1.958 | 13.455 | 1.815 | t(49) = 0.722 | 1.000 |
| Coverage | 7 | Noise vs Speech | 13.593 | 1.866 | 13.455 | 1.815 | t(49) = 0.137 | 1.000 |

Note. SE = standard error. Pairwise contrasts are Holm-adjusted estimated marginal mean comparisons for Condition from the prespecified Type III mixed repeated-measures model. The t ratio is reported for the first condition minus the second condition. *p < .05. **p < .01. ***p < .001.

**Additional Sensor-Level Omnibus and Interaction Analyses**

The following tables provide the Greenhouse–Geisser-corrected omnibus Condition and Group × Condition effects for the 15 sensor-level microstate models. Simple-condition effects are reported only for metrics with a significant Group × Condition interaction.

**Table S7**

*Sensor: Omnibus Condition and Group × Condition Effects*

| **Metric** | **Class** | **Effect** | **df** | **F** | **Value of p** | **partial η²** |
| --- | --- | --- | --- | --- | --- | --- |
| Duration | A | Condition | 1.82, 89.28 | 15.288 | < .001*** | 0.238 |
| Duration | A | Group x Condition | 3.64, 89.28 | 0.258 | .889 | 0.010 |
| Duration | B | Condition | 1.57, 77.13 | 13.982 | < .001*** | 0.222 |
| Duration | B | Group x Condition | 3.15, 77.13 | 0.027 | .995 | 0.001 |
| Duration | C | Condition | 1.66, 81.52 | 18.913 | < .001*** | 0.278 |
| Duration | C | Group x Condition | 3.33, 81.52 | 0.435 | .748 | 0.017 |
| Duration | D | Condition | 1.69, 82.76 | 23.558 | < .001*** | 0.325 |
| Duration | D | Group x Condition | 3.38, 82.76 | 0.345 | .815 | 0.014 |
| Duration | E | Condition | 1.69, 82.85 | 16.726 | < .001*** | 0.254 |
| Duration | E | Group x Condition | 3.38, 82.85 | 0.426 | .758 | 0.017 |
| Occurrence | A | Condition | 1.35, 66.34 | 14.665 | < .001*** | 0.230 |
| Occurrence | A | Group x Condition | 2.71, 66.34 | 2.662 | .061 | 0.098 |
| Occurrence | B | Condition | 1.41, 69.06 | 17.917 | < .001*** | 0.268 |
| Occurrence | B | Group x Condition | 2.82, 69.06 | 0.654 | .574 | 0.026 |
| Occurrence | C | Condition | 1.35, 66.33 | 18.295 | < .001*** | 0.272 |
| Occurrence | C | Group x Condition | 2.71, 66.33 | 0.200 | .878 | 0.008 |
| Occurrence | D | Condition | 1.64, 80.35 | 8.955 | < .001*** | 0.155 |
| Occurrence | D | Group x Condition | 3.28, 80.35 | 1.094 | .360 | 0.043 |
| Occurrence | E | Condition | 1.61, 78.70 | 14.840 | < .001*** | 0.232 |
| Occurrence | E | Group x Condition | 3.21, 78.70 | 3.614 | .015* | 0.129 |
| Coverage | A | Condition | 1.94, 95.22 | 0.385 | .675 | 0.008 |
| Coverage | A | Group x Condition | 3.89, 95.22 | 3.617 | .009** | 0.129 |
| Coverage | B | Condition | 1.74, 85.04 | 1.438 | .243 | 0.029 |
| Coverage | B | Group x Condition | 3.47, 85.04 | 0.255 | .883 | 0.010 |
| Coverage | C | Condition | 1.88, 91.94 | 0.457 | .622 | 0.009 |
| Coverage | C | Group x Condition | 3.75, 91.94 | 2.102 | .091 | 0.079 |
| Coverage | D | Condition | 1.92, 94.20 | 1.888 | .159 | 0.037 |
| Coverage | D | Group x Condition | 3.84, 94.20 | 0.606 | .653 | 0.024 |
| Coverage | E | Condition | 1.69, 82.74 | 3.133 | .057 | 0.060 |
| Coverage | E | Group x Condition | 3.38, 82.74 | 4.097 | .007** | 0.143 |

Note. Values are from Type III mixed repeated-measures ANOVA with Group (HC, MCS, VS/UWS) as the between-subjects factor and Condition (Rest, Noise, Speech) as the within-subject factor. The displayed df and p values are Greenhouse–Geisser corrected. Omnibus p values are not adjusted across the 15 models. N = 52. *p < .05. **p < .01. ***p < .001.

**Table S8**

*Sensor: Simple Condition Effects Within Group for Significant Interactions*

| **Metric** | **Class** | **Group** | **df** | **F** | **Value of p** | **partial η²** | **Interaction p** |
| --- | --- | --- | --- | --- | --- | --- | --- |
| Occurrence | E | HC | 1.92, 34.62 | 13.039 | < .001*** | 0.420 | .015* |
| Occurrence | E | MCS | 1.26, 18.84 | 10.217 | .003** | 0.405 | .015* |
| Occurrence | E | VS/UWS | 1.17, 18.67 | 0.134 | .757 | 0.008 | .015* |
| Coverage | A | HC | 1.99, 35.85 | 4.698 | .016* | 0.207 | .009** |
| Coverage | A | MCS | 1.77, 26.56 | 2.703 | .091 | 0.153 | .009** |
| Coverage | A | VS/UWS | 1.68, 26.86 | 0.969 | .379 | 0.057 | .009** |
| Coverage | E | HC | 1.29, 23.30 | 4.493 | .036* | 0.200 | .007** |
| Coverage | E | MCS | 1.61, 24.22 | 2.651 | .100 | 0.150 | .007** |
| Coverage | E | VS/UWS | 1.58, 25.35 | 4.365 | .031* | 0.214 | .007** |

Note. Simple Condition effects were evaluated separately within HC, MCS, and VS/UWS only when the corresponding Group × Condition interaction in Table S7 was significant. The displayed df and p values are Greenhouse–Geisser corrected. These omnibus simple-effect p values are unadjusted; Holm-adjusted pairwise decomposition is presented in Table S9. *p < .05. **p < .01. ***p < .001.

**Table S9**

*Sensor: Holm-Adjusted Simple Condition Pairwise Comparisons Within Group*

| **Metric** | **Class** | **Group** | **Comparison** | **Estimate** | **SE** | **df** | **t ratio** | **Value of p** |
| --- | --- | --- | --- | --- | --- | --- | --- | --- |
| Occurrence | E | HC | Rest vs Noise | 2.960 | 0.853 | 49.0 | 3.471 | .002** |
| Occurrence | E | HC | Rest vs Speech | 4.445 | 0.912 | 49.0 | 4.873 | < .001*** |
| Occurrence | E | HC | Noise vs Speech | 1.486 | 0.568 | 49.0 | 2.616 | .012* |
| Occurrence | E | MCS | Rest vs Noise | 2.291 | 0.929 | 49.0 | 2.466 | .034* |
| Occurrence | E | MCS | Rest vs Speech | 2.945 | 0.994 | 49.0 | 2.962 | .014* |
| Occurrence | E | MCS | Noise vs Speech | 0.654 | 0.619 | 49.0 | 1.057 | .296 |
| Occurrence | E | VS/UWS | Rest vs Noise | -0.119 | 0.901 | 49.0 | -0.132 | 1.000 |
| Occurrence | E | VS/UWS | Rest vs Speech | 0.314 | 0.964 | 49.0 | 0.325 | 1.000 |
| Occurrence | E | VS/UWS | Noise vs Speech | 0.433 | 0.601 | 49.0 | 0.721 | 1.000 |
| Coverage | A | HC | Rest vs Noise | 3.871 | 1.851 | 49.0 | 2.091 | .083 |
| Coverage | A | HC | Rest vs Speech | -0.941 | 1.859 | 49.0 | -0.506 | .615 |
| Coverage | A | HC | Noise vs Speech | -4.811 | 1.622 | 49.0 | -2.967 | .014* |
| Coverage | A | MCS | Rest vs Noise | -4.047 | 2.017 | 49.0 | -2.007 | .151 |
| Coverage | A | MCS | Rest vs Speech | -3.413 | 2.026 | 49.0 | -1.684 | .197 |
| Coverage | A | MCS | Noise vs Speech | 0.634 | 1.767 | 49.0 | 0.359 | .721 |
| Coverage | A | VS/UWS | Rest vs Noise | 1.541 | 1.957 | 49.0 | 0.788 | .869 |
| Coverage | A | VS/UWS | Rest vs Speech | 2.876 | 1.966 | 49.0 | 1.463 | .449 |
| Coverage | A | VS/UWS | Noise vs Speech | 1.335 | 1.714 | 49.0 | 0.779 | .869 |
| Coverage | E | HC | Rest vs Noise | -0.396 | 1.693 | 49.0 | -0.234 | .816 |
| Coverage | E | HC | Rest vs Speech | 5.035 | 2.396 | 49.0 | 2.101 | .082 |
| Coverage | E | HC | Noise vs Speech | 5.431 | 1.880 | 49.0 | 2.889 | .017* |
| Coverage | E | MCS | Rest vs Noise | 2.314 | 1.845 | 49.0 | 1.254 | .406 |
| Coverage | E | MCS | Rest vs Speech | 4.959 | 2.611 | 49.0 | 1.899 | .190 |
| Coverage | E | MCS | Noise vs Speech | 2.645 | 2.049 | 49.0 | 1.291 | .406 |
| Coverage | E | VS/UWS | Rest vs Noise | -5.956 | 1.790 | 49.0 | -3.328 | .005** |
| Coverage | E | VS/UWS | Rest vs Speech | -4.896 | 2.533 | 49.0 | -1.932 | .118 |
| Coverage | E | VS/UWS | Noise vs Speech | 1.060 | 1.988 | 49.0 | 0.533 | .596 |

Note. Estimated marginal-mean contrasts are reported as the first condition minus the second condition. Holm adjustment was applied to the three condition contrasts separately within each Metric × Class × Group family. Only interactions significant in Table S7 were decomposed. *p < .05. **p < .01. ***p < .001.

Interpretive summary. The significant interactions indicate group-dependent modulation across conditions. Class E occurrence decreased from rest to auditory stimulation in HC and MCS but remained stable in VS/UWS. Coverage effects were more selective: HC showed higher class A coverage during speech than noise and higher class E coverage during noise than speech, whereas VS/UWS showed higher class E coverage during noise than at rest. These findings should therefore be interpreted as group-specific simple effects rather than pooled condition differences.

**Speech-condition pooling**

Native and non-native speech were combined into a single Speech level before inferential analysis. The analyses reported here therefore address rest, noise, and pooled speech and do not test differences between the two speech conditions or establish their equivalence.

**Table S10** Sensor-level microstate metrics (duration, occurrence, and coverage) for HC, VS/UWS, and MCS patients.

| Variable | **MS** | **HC M(SE)** | **MCS M(SE)** | **VS M(SE)** | **F(df1, df2) (p)** |
| --- | --- | --- | --- | --- | --- |
| Duration | A | 29.565 (1.179) | 40.932 (2.090) | 42.882 (2.570) | F(2, 49) = 13.848 (< .001***) |
|  | B | 28.912 (0.855) | 40.743 (2.343) | 41.061 (2.713) | F(2, 49) = 11.912 (< .001***) |
|  | C | 32.309 (1.091) | 41.431 (2.229) | 42.925 (2.542) | F(2, 49) = 8.743 (< .001***) |
|  | D | 29.327 (0.955) | 37.348 (1.810) | 40.805 (2.993) | F(2, 49) = 8.713 (< .001***) |
|  | E | 29.112 (1.025) | 40.212 (2.414) | 43.397 (2.494) | F(2, 49) = 14.495 (< .001***) |
| Occurrence Rate | A | 6.784 (0.429) | 5.741 (0.419) | 5.681 (0.388) | F(2, 49) = 2.334 (.108) |
|  | B | 7.406 (0.366) | 5.726 (0.409) | 5.724 (0.473) | F(2, 49) = 5.684 (.006**) |
|  | C | 8.039 (0.357) | 5.841 (0.390) | 5.468 (0.488) | F(2, 49) = 11.84 (< .001***) |
|  | D | 6.74 (0.473) | 4.967 (0.408) | 4.631 (0.383) | F(2, 49) = 7.311 (.002**) |
|  | E | 6.673 (0.435) | 5.073 (0.312) | 4.931 (0.414) | F(2, 49) = 6.175 (.004**) |
| Coverage | A | 18.36 (1.001) | 21.635 (0.773) | 21.855 (0.712) | F(2, 49) = 5.452 (.007**) |
|  | B | 20.292 (0.693) | 21.234 (0.813) | 20.943 (0.767) | F(2, 49) = 0.419 (.660) |
|  | C | 24.684 (1.063) | 21.777 (0.777) | 20.783 (1.122) | F(2, 49) = 4.182 (.021*) |
|  | D | 18.458 (0.922) | 16.72 (0.909) | 16.952 (1.088) | F(2, 49) = 0.968 (.387) |
|  | E | 18.206 (0.961) | 18.634 (0.984) | 19.467 (0.957) | F(2, 49) = 0.448 (.641) |

Note. M (SE) summarizes each participant’s mean across Rest, Noise, and Speech; SE is the between-participant sample SD divided by the square root of the group sample size. These descriptive SEs differ from the model-based estimated marginal-mean SEs in Table S1. Dur. = duration; Occ. = occurrence; Cov. = coverage. The last column reports the Type III Group main effect as F(2, 49) (p). Group is a between-subject effect and does not require Greenhouse–Geisser correction. Corresponding partial η² values are reported in Table S13. Omnibus p values are not adjusted across classes or metrics. *p < .05. **p < .01. ***p < .001.

**Table S11** Post hoc comparisons of sensor-level microstate metrics between VS/UWS and MCS patients.

| Variable | **MS** | **MCS M(SE)** | **VS M(SE)** | t(df) (p) |
| --- | --- | --- | --- | --- |
| Duration | A | 40.932 (2.090) | 42.882 (2.570) | t(49) = −0.68 (.500) |
|  | B | 40.743 (2.343) | 41.061 (2.713) | t(49) = −0.107 (.915) |
|  | C | 41.431 (2.229) | 42.925 (2.542) | t(49) = −0.518 (.607) |
|  | D | 37.348 (1.810) | 40.805 (2.993) | t(49) = −1.169 (.248) |
|  | E | 40.212 (2.414) | 43.397 (2.494) | t(49) = −1.09 (.281) |
| Occurrence Rate | A | 5.741 (0.419) | 5.681 (0.388) | t(49) = 0.1 (.921) |
|  | B | 5.726 (0.409) | 5.724 (0.473) | t(49) = 0.002 (.998) |
|  | C | 5.841 (0.390) | 5.468 (0.488) | t(49) = 0.623 (.536) |
|  | D | 4.967 (0.408) | 4.631 (0.383) | t(49) = 0.54 (.592) |
|  | E | 5.073 (0.312) | 4.931 (0.414) | t(49) = 0.247 (.806) |
| Coverage | A | 21.635 (0.773) | 21.855 (0.712) | t(49) = −0.177 (.860) |
|  | B | 21.234 (0.813) | 20.943 (0.767) | t(49) = 0.266 (1.000) |
|  | C | 21.777 (0.777) | 20.783 (1.122) | t(49) = 0.676 (.502) |
|  | D | 16.72 (0.909) | 16.952 (1.088) | t(49) = −0.164 (.871) |
|  | E | 18.634 (0.984) | 19.467 (0.957) | t(49) = −0.593 (1.000) |

Note. M (SE) summarizes each participant’s mean across Rest, Noise, and Speech; SE is the between-participant sample SD divided by the square root of the group sample size. These descriptive SEs differ from the model-based estimated marginal-mean SEs in Table S1. Dur. = duration; Occ. = occurrence; Cov. = coverage. The last column reports t(49) (Holm-adjusted p) for MCS minus VS/UWS from the full three-group model. Holm correction covers the three group contrasts separately for each class-by-metric model. Confirmatory interpretation requires a significant corresponding Group main effect; other contrasts are exploratory. *p < .05. **p < .01. ***p < .001.

**Table S12**

*Clinical characteristics of the patient groups*

| **Characteristic** | **MCS (n = 16)** | **VS/UWS (n = 17)** | **Test statistic** | **p** |
| --- | --- | --- | --- | --- |
| Etiology, TBI/stroke, n | 8/8 | 8/9 | Fisher | 1.000 |
| Time since injury, months, median [Q1, Q3] | 3.75 [1.50, 6.13] | 4.00 [2.00, 7.00] | W = 119.00 | .550 |
| Sex, male/female, n | 8/8 | 11/6 | Fisher | .491 |
| Age, years, mean (SD) | 54.19 (13.17) | 50.59 (10.90) | t(29.19) = 0.85 | .401 |

Note. TBI, traumatic brain injury; SD, standard deviation; Q1 and Q3, the 25th and 75th percentiles. Etiology and sex were compared using two-sided Fisher exact tests; time since injury using a Wilcoxon rank-sum test with continuity correction; and age using a Welch t test. The age comparison describes baseline characteristics and does not adjust EEG results. Non-significant comparisons do not establish equivalence or exclude residual confounding. No inferential comparison involving HC is included.

**Table S13**

*Sensor-level microstate mixed ANOVA*

| **Metric** | **Class** | **Effect** | **F(df1, df2)** | **p** | **Partial η²** |
| --- | --- | --- | --- | --- | --- |
| Duration (ms) | MS-A | Group | F(2, 49) = 13.848 | < .001 | 0.361 |
| Duration (ms) | MS-A | Condition | F(1.822, 89.279) = 15.288 | < .001 | 0.238 |
| Duration (ms) | MS-A | Group × Condition | F(3.644, 89.279) = 0.258 | .889 | 0.010 |
| Duration (ms) | MS-B | Group | F(2, 49) = 11.912 | < .001 | 0.327 |
| Duration (ms) | MS-B | Condition | F(1.574, 77.129) = 13.982 | < .001 | 0.222 |
| Duration (ms) | MS-B | Group × Condition | F(3.148, 77.129) = 0.027 | .995 | 0.001 |
| Duration (ms) | MS-C | Group | F(2, 49) = 8.743 | < .001 | 0.263 |
| Duration (ms) | MS-C | Condition | F(1.664, 81.520) = 18.913 | < .001 | 0.278 |
| Duration (ms) | MS-C | Group × Condition | F(3.327, 81.520) = 0.435 | .748 | 0.017 |
| Duration (ms) | MS-D | Group | F(2, 49) = 8.713 | < .001 | 0.262 |
| Duration (ms) | MS-D | Condition | F(1.689, 82.759) = 23.558 | < .001 | 0.325 |
| Duration (ms) | MS-D | Group × Condition | F(3.378, 82.759) = 0.345 | .815 | 0.014 |
| Duration (ms) | MS-E | Group | F(2, 49) = 14.495 | < .001 | 0.372 |
| Duration (ms) | MS-E | Condition | F(1.691, 82.852) = 16.726 | < .001 | 0.254 |
| Duration (ms) | MS-E | Group × Condition | F(3.382, 82.852) = 0.426 | .758 | 0.017 |
| Coverage (%) | MS-A | Group | F(2, 49) = 5.452 | .007 | 0.182 |
| Coverage (%) | MS-A | Condition | F(1.943, 95.221) = 0.385 | .675 | 0.008 |
| Coverage (%) | MS-A | Group × Condition | F(3.887, 95.221) = 3.617 | .009 | 0.129 |
| Coverage (%) | MS-B | Group | F(2, 49) = 0.419 | .660 | 0.017 |
| Coverage (%) | MS-B | Condition | F(1.735, 85.037) = 1.438 | .243 | 0.029 |
| Coverage (%) | MS-B | Group × Condition | F(3.471, 85.037) = 0.255 | .883 | 0.010 |
| Coverage (%) | MS-C | Group | F(2, 49) = 4.182 | .021 | 0.146 |
| Coverage (%) | MS-C | Condition | F(1.876, 91.942) = 0.457 | .622 | 0.009 |
| Coverage (%) | MS-C | Group × Condition | F(3.753, 91.942) = 2.102 | .091 | 0.079 |
| Coverage (%) | MS-D | Group | F(2, 49) = 0.968 | .387 | 0.038 |
| Coverage (%) | MS-D | Condition | F(1.922, 94.195) = 1.888 | .159 | 0.037 |
| Coverage (%) | MS-D | Group × Condition | F(3.845, 94.195) = 0.606 | .653 | 0.024 |
| Coverage (%) | MS-E | Group | F(2, 49) = 0.448 | .641 | 0.018 |
| Coverage (%) | MS-E | Condition | F(1.689, 82.738) = 3.133 | .057 | 0.060 |
| Coverage (%) | MS-E | Group × Condition | F(3.377, 82.738) = 4.097 | .007 | 0.143 |
| Occurrence (Hz) | MS-A | Group | F(2, 49) = 2.334 | .108 | 0.087 |
| Occurrence (Hz) | MS-A | Condition | F(1.354, 66.342) = 14.665 | < .001 | 0.230 |
| Occurrence (Hz) | MS-A | Group × Condition | F(2.708, 66.342) = 2.662 | .061 | 0.098 |
| Occurrence (Hz) | MS-B | Group | F(2, 49) = 5.684 | .006 | 0.188 |
| Occurrence (Hz) | MS-B | Condition | F(1.409, 69.060) = 17.917 | < .001 | 0.268 |
| Occurrence (Hz) | MS-B | Group × Condition | F(2.819, 69.060) = 0.654 | .574 | 0.026 |
| Occurrence (Hz) | MS-C | Group | F(2, 49) = 11.840 | < .001 | 0.326 |
| Occurrence (Hz) | MS-C | Condition | F(1.354, 66.335) = 18.295 | < .001 | 0.272 |
| Occurrence (Hz) | MS-C | Group × Condition | F(2.708, 66.335) = 0.200 | .878 | 0.008 |
| Occurrence (Hz) | MS-D | Group | F(2, 49) = 7.311 | .002 | 0.230 |
| Occurrence (Hz) | MS-D | Condition | F(1.640, 80.352) = 8.955 | < .001 | 0.155 |
| Occurrence (Hz) | MS-D | Group × Condition | F(3.280, 80.352) = 1.094 | .360 | 0.043 |
| Occurrence (Hz) | MS-E | Group | F(2, 49) = 6.175 | .004 | 0.201 |
| Occurrence (Hz) | MS-E | Condition | F(1.606, 78.698) = 14.840 | < .001 | 0.232 |
| Occurrence (Hz) | MS-E | Group × Condition | F(3.212, 78.698) = 3.614 | .015 | 0.129 |

Note. N = 52 (HC = 19, MCS = 16, VS/UWS = 17). Type III mixed repeated-measures ANOVA with Noise, Speech and REST as within-subject conditions. Condition and Group × Condition effects use GG-corrected degrees of freedom and p values. Group is a between-subject effect and does not require GG correction. Partial η² denotes partial eta squared. Omnibus tests are not additionally adjusted across classes or metrics. Uncorrected p values, original degrees of freedom and GG epsilon are retained in Supplementary_omnibus_statistics.csv.

**Table S14**

*Source-level microstate mixed ANOVA*

| **Metric** | **Class** | **Effect** | **F(df1, df2)** | **p** | **Partial η²** |
| --- | --- | --- | --- | --- | --- |
| Duration (ms) | MS1 | Group | F(2, 49) = 5.050 | .010 | 0.171 |
| Duration (ms) | MS1 | Condition | F(1.297, 63.540) = 0.805 | .403 | 0.016 |
| Duration (ms) | MS1 | Group × Condition | F(2.593, 63.540) = 0.767 | .500 | 0.030 |
| Duration (ms) | MS2 | Group | F(2, 49) = 8.474 | < .001 | 0.257 |
| Duration (ms) | MS2 | Condition | F(1.629, 79.809) = 1.728 | .189 | 0.034 |
| Duration (ms) | MS2 | Group × Condition | F(3.258, 79.809) = 0.571 | .649 | 0.023 |
| Duration (ms) | MS3 | Group | F(2, 49) = 11.519 | < .001 | 0.320 |
| Duration (ms) | MS3 | Condition | F(1.739, 85.233) = 0.730 | .467 | 0.015 |
| Duration (ms) | MS3 | Group × Condition | F(3.479, 85.233) = 1.475 | .222 | 0.057 |
| Duration (ms) | MS4 | Group | F(2, 49) = 8.987 | < .001 | 0.268 |
| Duration (ms) | MS4 | Condition | F(1.685, 82.551) = 1.148 | .315 | 0.023 |
| Duration (ms) | MS4 | Group × Condition | F(3.369, 82.551) = 0.241 | .887 | 0.010 |
| Duration (ms) | MS5 | Group | F(2, 49) = 9.041 | < .001 | 0.270 |
| Duration (ms) | MS5 | Condition | F(1.188, 58.197) = 0.538 | .496 | 0.011 |
| Duration (ms) | MS5 | Group × Condition | F(2.375, 58.197) = 0.668 | .542 | 0.027 |
| Duration (ms) | MS6 | Group | F(2, 49) = 6.460 | .003 | 0.209 |
| Duration (ms) | MS6 | Condition | F(1.682, 82.429) = 0.273 | .723 | 0.006 |
| Duration (ms) | MS6 | Group × Condition | F(3.364, 82.429) = 0.705 | .567 | 0.028 |
| Duration (ms) | MS7 | Group | F(2, 49) = 12.372 | < .001 | 0.336 |
| Duration (ms) | MS7 | Condition | F(1.222, 59.875) = 0.075 | .833 | 0.002 |
| Duration (ms) | MS7 | Group × Condition | F(2.444, 59.875) = 1.913 | .148 | 0.072 |
| Coverage (%) | MS1 | Group | F(2, 49) = 0.891 | .417 | 0.035 |
| Coverage (%) | MS1 | Condition | F(1.508, 73.906) = 0.446 | .586 | 0.009 |
| Coverage (%) | MS1 | Group × Condition | F(3.017, 73.906) = 0.598 | .619 | 0.024 |
| Coverage (%) | MS2 | Group | F(2, 49) = 0.086 | .918 | 0.004 |
| Coverage (%) | MS2 | Condition | F(1.252, 61.350) = 0.578 | .487 | 0.012 |
| Coverage (%) | MS2 | Group × Condition | F(2.504, 61.350) = 0.774 | .492 | 0.031 |
| Coverage (%) | MS3 | Group | F(2, 49) = 6.880 | .002 | 0.219 |
| Coverage (%) | MS3 | Condition | F(1.216, 59.601) = 0.414 | .562 | 0.008 |
| Coverage (%) | MS3 | Group × Condition | F(2.433, 59.601) = 0.898 | .430 | 0.035 |
| Coverage (%) | MS4 | Group | F(2, 49) = 4.443 | .017 | 0.154 |
| Coverage (%) | MS4 | Condition | F(1.428, 69.993) = 1.610 | .211 | 0.032 |
| Coverage (%) | MS4 | Group × Condition | F(2.857, 69.993) = 0.318 | .803 | 0.013 |
| Coverage (%) | MS5 | Group | F(2, 49) = 1.320 | .276 | 0.051 |
| Coverage (%) | MS5 | Condition | F(1.509, 73.953) = 0.558 | .527 | 0.011 |
| Coverage (%) | MS5 | Group × Condition | F(3.018, 73.953) = 0.877 | .458 | 0.035 |
| Coverage (%) | MS6 | Group | F(2, 49) = 2.165 | .126 | 0.081 |
| Coverage (%) | MS6 | Condition | F(1.640, 80.354) = 0.576 | .531 | 0.012 |
| Coverage (%) | MS6 | Group × Condition | F(3.280, 80.354) = 1.065 | .372 | 0.042 |
| Coverage (%) | MS7 | Group | F(2, 49) = 1.041 | .361 | 0.041 |
| Coverage (%) | MS7 | Condition | F(1.478, 72.427) = 0.380 | .622 | 0.008 |
| Coverage (%) | MS7 | Group × Condition | F(2.956, 72.427) = 0.860 | .465 | 0.034 |
| Occurrence (Hz) | MS1 | Group | F(2, 49) = 10.083 | < .001 | 0.292 |
| Occurrence (Hz) | MS1 | Condition | F(1.611, 78.931) = 0.285 | .705 | 0.006 |
| Occurrence (Hz) | MS1 | Group × Condition | F(3.222, 78.931) = 0.352 | .802 | 0.014 |
| Occurrence (Hz) | MS2 | Group | F(2, 49) = 5.636 | .006 | 0.187 |
| Occurrence (Hz) | MS2 | Condition | F(1.355, 66.399) = 0.218 | .716 | 0.004 |
| Occurrence (Hz) | MS2 | Group × Condition | F(2.710, 66.399) = 0.794 | .490 | 0.031 |
| Occurrence (Hz) | MS3 | Group | F(2, 49) = 5.498 | .007 | 0.183 |
| Occurrence (Hz) | MS3 | Condition | F(1.378, 67.543) = 2.290 | .126 | 0.045 |
| Occurrence (Hz) | MS3 | Group × Condition | F(2.757, 67.543) = 0.844 | .466 | 0.033 |
| Occurrence (Hz) | MS4 | Group | F(2, 49) = 9.235 | < .001 | 0.274 |
| Occurrence (Hz) | MS4 | Condition | F(1.689, 82.764) = 1.226 | .294 | 0.024 |
| Occurrence (Hz) | MS4 | Group × Condition | F(3.378, 82.764) = 0.262 | .874 | 0.011 |
| Occurrence (Hz) | MS5 | Group | F(2, 49) = 9.052 | < .001 | 0.270 |
| Occurrence (Hz) | MS5 | Condition | F(1.628, 79.769) = 0.448 | .600 | 0.009 |
| Occurrence (Hz) | MS5 | Group × Condition | F(3.256, 79.769) = 1.657 | .179 | 0.063 |
| Occurrence (Hz) | MS6 | Group | F(2, 49) = 10.615 | < .001 | 0.302 |
| Occurrence (Hz) | MS6 | Condition | F(1.546, 75.735) = 0.682 | .472 | 0.014 |
| Occurrence (Hz) | MS6 | Group × Condition | F(3.091, 75.735) = 0.411 | .751 | 0.017 |
| Occurrence (Hz) | MS7 | Group | F(2, 49) = 1.533 | .226 | 0.059 |
| Occurrence (Hz) | MS7 | Condition | F(1.790, 87.693) = 0.339 | .690 | 0.007 |
| Occurrence (Hz) | MS7 | Group × Condition | F(3.579, 87.693) = 0.450 | .751 | 0.018 |

Note. N = 52 (HC = 19, MCS = 16, VS/UWS = 17). Type III mixed repeated-measures ANOVA with Noise, Speech and REST as within-subject conditions. Condition and Group × Condition effects use GG-corrected degrees of freedom and p values. Group is a between-subject effect and does not require GG correction. Partial η² denotes partial eta squared. Omnibus tests are not additionally adjusted across classes or metrics. Uncorrected p values, original degrees of freedom and GG epsilon are retained in Supplementary_omnibus_statistics.csv.

**Table S15**

*Sphericity tests and GG epsilon*

| **Level** | **Class** | **Metric** | **Mauchly W** | **p** | **GG ε** |
| --- | --- | --- | --- | --- | --- |
| Sensor | MS-A | Coverage (%) | 0.971 | .491 | 0.972 |
| Sensor | MS-A | Duration (ms) | 0.902 | .085 | 0.911 |
| Sensor | MS-A | Occurrence (Hz) | 0.523 | < .001 | 0.677 |
| Sensor | MS-B | Coverage (%) | 0.848 | .019 | 0.868 |
| Sensor | MS-B | Duration (ms) | 0.729 | < .001 | 0.787 |
| Sensor | MS-B | Occurrence (Hz) | 0.581 | < .001 | 0.705 |
| Sensor | MS-C | Coverage (%) | 0.934 | .195 | 0.938 |
| Sensor | MS-C | Duration (ms) | 0.798 | .004 | 0.832 |
| Sensor | MS-C | Occurrence (Hz) | 0.523 | < .001 | 0.677 |
| Sensor | MS-D | Coverage (%) | 0.960 | .372 | 0.961 |
| Sensor | MS-D | Duration (ms) | 0.816 | .008 | 0.844 |
| Sensor | MS-D | Occurrence (Hz) | 0.780 | .003 | 0.820 |
| Sensor | MS-E | Coverage (%) | 0.816 | .007 | 0.844 |
| Sensor | MS-E | Duration (ms) | 0.817 | .008 | 0.845 |
| Sensor | MS-E | Occurrence (Hz) | 0.755 | .001 | 0.803 |
| Source | MS1 | Coverage (%) | 0.674 | < .001 | 0.754 |
| Source | MS1 | Duration (ms) | 0.458 | < .001 | 0.648 |
| Source | MS1 | Occurrence (Hz) | 0.758 | .001 | 0.805 |
| Source | MS2 | Coverage (%) | 0.403 | < .001 | 0.626 |
| Source | MS2 | Duration (ms) | 0.772 | .002 | 0.814 |
| Source | MS2 | Occurrence (Hz) | 0.524 | < .001 | 0.678 |
| Source | MS3 | Coverage (%) | 0.356 | < .001 | 0.608 |
| Source | MS3 | Duration (ms) | 0.850 | .020 | 0.870 |
| Source | MS3 | Occurrence (Hz) | 0.549 | < .001 | 0.689 |
| Source | MS4 | Coverage (%) | 0.600 | < .001 | 0.714 |
| Source | MS4 | Duration (ms) | 0.813 | .007 | 0.842 |
| Source | MS4 | Occurrence (Hz) | 0.816 | .008 | 0.845 |
| Source | MS5 | Coverage (%) | 0.675 | < .001 | 0.755 |
| Source | MS5 | Duration (ms) | 0.316 | < .001 | 0.594 |
| Source | MS5 | Occurrence (Hz) | 0.771 | .002 | 0.814 |
| Source | MS6 | Coverage (%) | 0.780 | .003 | 0.820 |
| Source | MS6 | Duration (ms) | 0.811 | .007 | 0.841 |
| Source | MS6 | Occurrence (Hz) | 0.706 | < .001 | 0.773 |
| Source | MS7 | Coverage (%) | 0.647 | < .001 | 0.739 |
| Source | MS7 | Duration (ms) | 0.363 | < .001 | 0.611 |
| Source | MS7 | Occurrence (Hz) | 0.882 | .0498 | 0.895 |

Note. One sphericity test is reported per class-by-metric model; Condition and the interaction are not counted as separate diagnostics. Mauchly p < .05 in 32 of 36 models, and GG epsilon ranged from .594 to .972. GG-corrected inference is reported consistently for all condition-related effects, without selecting correction rules according to significance.
